# Interpretable Machine Learning Approaches for Forecasting and Predicting Air Pollution: A Systematic Review

**DOI:** 10.4209/aaqr.230151

**Published:** 2023-11-30

**Authors:** Anass Houdou, Imad El Badisy, Kenza Khomsi, Sammila Andrade Abdala, Fayez Abdulla, Houda Najmi, Majdouline Obtel, Lahcen Belyamani, Azeddine Ibrahimi, Mohamed Khalis

**Affiliations:** 1 Mohammed VI Center for Research & Innovation, Rabat, Morocco; 2 International School of Public Health, Mohammed VI University of Sciences and Health, Casablanca, Morocco; 3 Inserm UMR912 Sciences Economiques & Sociales de la Santé & Traitement de l'Information Médicale (SESSTIM), Marseille, France; 4 General Directorate of Meteorology, Mohammed VI University of Sciences and Health, Casablanca, Morocco; 5 General Directorate of Meteorology, Casablanca, Morocco; 6 Civil Engineering Department, Jordan University of Science and Technology, Irbid 22120, Jordan; 7 Laboratory of Biostatistics, Clinical, and Epidemiological Research, & Laboratory of Community Health (Public Health, Preventive Medicine and Hygiene), Department of Public Health, Faculty of Medicine and Pharmacy, Mohammed V University in Rabat, Rabat, Morocco; 8 Mohammed VI University of Sciences and Health, Casablanca, Morocco; 9 Faculty of Medicine and Pharmacy, Mohammed V University in Rabat, Rabat, Morocco; 10 Higher Institute of Nursing Professions and Technical Health, Rabat, Morocco

**Keywords:** Air quality prediction, Deep learning, Supervised learning

## Abstract

Many studies use machine learning to predict atmospheric pollutant levels, prioritizing accuracy over interpretability. This systematic review will focus on reviewing studies that have utilized interpretable machine learning models to enhance interpretability while maintaining high accuracy for air pollution prediction. The search terms "air pollution," "machine learning," and "interpretability" were used to identify relevant studies published between 2011 and 2023 from PubMed, Scopus, Web of Science, Science Direct, and JuSER. The included studies were assessed for quality based on an ecological checklist for maximizing reproducibility of ecological niche models. Among the 5,396 identified studies, 480 focused on air pollution prediction, with 56 providing model interpretations. Among the studies, 20 methods were identified: 8 model-agnostic methods, 4 model-specific methods, and 8 hybrid models. Shapley additive explanations was the most commonly used method (46.4%), followed by partial dependence plots (17.4%), both of which are model-agnostic methods. These methods identify important atmospheric features, enhancing researchers' understanding and making machine learning outcomes more accessible to non-experts. This can enhance prediction and prevention of adverse weather events and air pollution, benefiting public health.

## 1 INTRODUCTION

Air pollution has severe global implications for human health, the economy, and the environment. Factors like industrial and agricultural activities, urbanization, transportation, and infrastructure contribute significantly to air pollution in urban areas (Zaini et al., 2022). Exposure to air pollutants can result in serious health problems, including respiratory issues, lung cancer, and heart disease. Studies have established a strong link between air pollution and mortality, with particulate matter and sulfur dioxide showing notable correlations (Chen et al., 2019; Yan et al., 2019; O’Brien et al., 2023). Air pollution has also been associated with lung function issues in children (Wang et al., 2015), increased lung cancer risk (Hart et al., 2015; Puett et al., 2014), and mortality due to cardiovascular disease (Chen et al., 2020). Ozone concentrations have been linked to respiratory disease, asthma, cardiovascular disease, neoplasms, and other health problems (Li et al., 2021; Malig et al., 2016; Malley et al., 2017).

Monitoring air quality through measurement stations is valuable, but predicting air pollution in areas without monitoring stations is crucial for implementing preventive measures. Machine learning, a subfield of artificial intelligence, has demonstrated its effectiveness in environmental research, allowing for accurate air quality prediction and forecasting (Wu and Lin, 2019). There have been numerous studies conducted on developing air quality prediction and forecasting models using machine learning to control air pollution. These studies have led to a considerable number of reviews and systematic reviews on the application of machine learning in predicting air quality. Advanced techniques like decision trees (DT), support vector machines (SVMs), and multilayer perceptron (MLP) have been demonstrated for predicting ozone concentrations (Yafouz et al., 2021). Deep learning algorithms and various other forecasting algorithms, along with data processing, ensemble learning, and metaheuristic optimization methods, have been summarized and discussed by Zaini et al. (2022). The commonly used AI-based techniques for air pollution forecasting have also been comprehensively reviewed by Masood and Ahmad (2021).

Although these reviews offer insights into the application of machine learning for air quality prediction, none of them discuss the most crucial aspect of interpretability. Interpretability refers to the extent to which a human can comprehend the reason behind a decision (Molnar, 2022). In the early stages, traditional statistical models such us linear regression and generalized additive model (GAM) were primarily used for air pollution prediction modeling, offering good interpretability but with lower model accuracy. To enhance accuracy, researchers started adopting more complex machine learning techniques like random forest (RF), support vector machines (SVM), and deep learning. These methods effectively capture air pollution distribution and improve prediction accuracy; however, they sacrifice interpretability and fail to provide explanations for their predictions. This presents a significant challenge for decision-makers who require reliable and comprehensible information to make informed decisions for air pollution control.

With the growing interest in interpretable machine learning models in recent years (Molnar et al., 2020), researchers have been exploring their application in air pollution predictions, aiming to improve interpretability while maintaining high accuracy (Ahmad et al., 2022; Shi et al., 2023; Yang et al., 2022b; Jovanovic et al., 2023). Given the increasing interest of policymakers in comprehending model decisions and the lack of comprehensive reviews on this topic, there is a clear need for a review that summarizes the use of interpretable machine learning models in the interpretation and explanation of air pollution predictions.

The objective of this systematic review is to concentrate on papers that utilize interpretable machine learning models for air pollution prediction and forecasting, aiming to improve model interpretability while maintaining high model accuracy and to showcase methods for explaining the most challenging, uninterpretable models.

## 2 METHODS

### 2.1 Protocol and Registration

This systematic review was conducted in accordance with the preferred reporting items for systematic reviews and meta-analyses (PRISMA) statement (Page et al., 2021). It was registered and published on the international prospective register of systematic reviews (PROSPERO) on 17 March 2022, with the registration number CRD42022319034, and is accessible via the following link: https://www.crd.york.ac.uk/prospero/display_record.php?ID=CRD42022319034.

### 2.2 Information Sources

Our search spanned five databases: Scopus, PubMed, Science Direct, Web of Science, and JuSER, the reference system for publications from the jülich research center (JuSER, 2023). To determine the search terms, we created a sample set of papers focused on air quality prediction using interpretable machine learning. This involved conducting preliminary "scoping searches" on Google Scholar and reviewing previous studies and systematic reviews in the field. From these papers, we identified key words, synonyms, and related terms.

### 2.3 Search Strategy

Our research strategy involved using three main concepts: "air pollution," "machine learning," and "interpretability," along with their synonyms and related terms, as search terms. We focused on identifying relevant studies published in English that included these terms in the titles, abstracts, or keywords. To ensure comprehensive coverage, we limited our search to articles published between 2011 and 2022, as the field of interpretable machine learning gained popularity around 2015 (Molnar et al., 2020). Subject area restrictions such as "Environmental Science," "Engineering," and "Computer Science" were applied. A broader strategy was employed in PubMed, Science Direct, and JuSER to capture any relevant papers. For completeness, we conducted additional searches for studies published between 2022 and 2023 and included them in the review. The details of our search strategies are shown in the Supplemental Material, Table S1.

### 2.4 Eligibility Criteria

Articles were eligible for inclusion in this systematic review if they interpreted the machine learning algorithms used to predict and forecast outdoor air pollution, with a focus on improving interpretability while maintaining high accuracy. Inclusion criteria encompassed articles published in scientific journals between January 2011 and February 2023, and written in English. Studies that used interpretable models with low accuracy or failed to improve interpretability while enhancing accuracy were excluded. Additionally, studies without abstracts or those for which the full text was unavailable were excluded.

### 2.5 Selection Process

Zotero reference manager (Zotero, 2023) was used to organize and detect duplicate references. Further duplicates were eliminated during the screening. The first author (AH) screened for relevant studies identified by the search, and the second author (IE) checked decisions. Titles and abstracts were reviewed, excluding articles that didn't use interpretable machine learning for air quality prediction or lacked clear interpretations. Full texts of potentially eligible papers were independently reviewed by both authors to determine inclusion. Discrepancies were resolved through discussion or with other authors (KK, MK) if needed. Eligible articles were identified using the PRISMA flow diagram.

### 2.6 Data Collection Process and Data Items

The first author (AH) extracted data from the selected articles, and decisions were checked with the second author (IE). Any discrepancies or disagreements regarding relevant data were resolved through consensus via discussion between the two, or with involvement from other authors (KK, MK) if needed. The selected articles underwent thorough analysis, and the following information was extracted from each: reference, study area, air pollutants of interest, sample size, study objective, model or approach used for prediction, model performance, interpretation methods, and interpretation results.

### 2.7 Study Risk of Bias Assessment

To assess bias risk in the included studies, we used an updated ecological checklist by Feng et al. (2019). We adapted the checklist by including components to evaluate the title, abstract, results, and discussion for minimizing bias risk. Additional components were added to address machine learning interpretability. The complete checklist is available in the Supplemental Material, Table S2. Each checklist item was answered with 'Yes', 'No', or 'Not Applicable' (NA). The quality of each article was determined based on the number of 'Yes' or 'No' answers. Articles with mostly 'Yes' answers were considered high quality, while those with mostly 'No' answers were deemed low quality. Articles with an equal number of 'Yes' and 'No' answers were considered medium quality. The first author (AH) evaluated study quality, and the second author (IE) checked decisions. Any discrepancies or disagreements were resolved through consensus via discussion between the two, or with involvement from other authors (KK, MK) if needed.

## 3 RESULTS

### 3.1 Study Selection

**Fig. 1.**
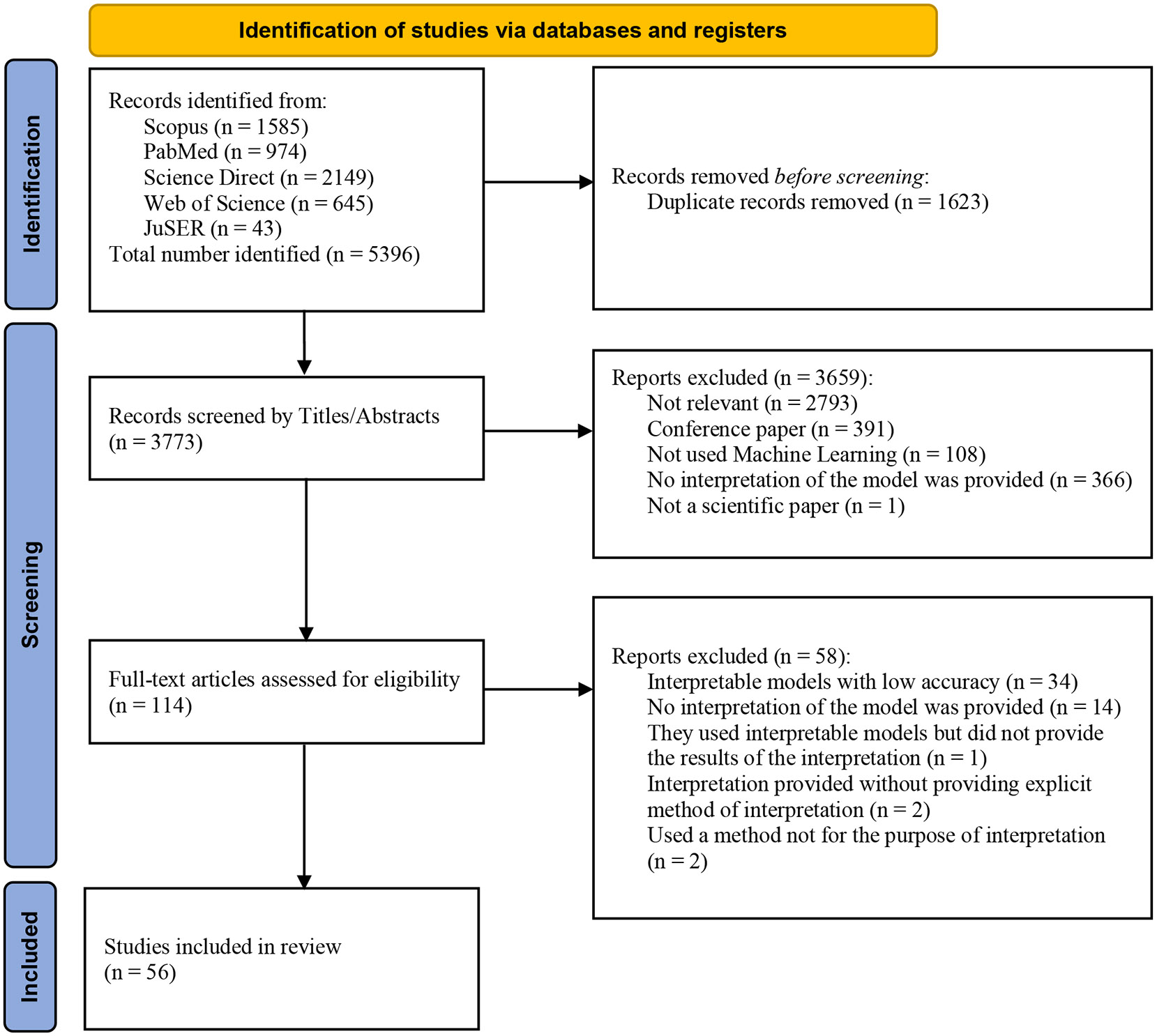
PRISMA diagram flow for studies selection.

We identified 5,396 studies, with 480 of them focused on air pollution prediction using machine learning. After removing duplicates and irrelevant studies, we only reviewed 114 studies in full. Out of these, 56 studies met our criteria and were included in the review (Fig. 1).

### 3.2 Study Characteristics

**Fig. 2.**
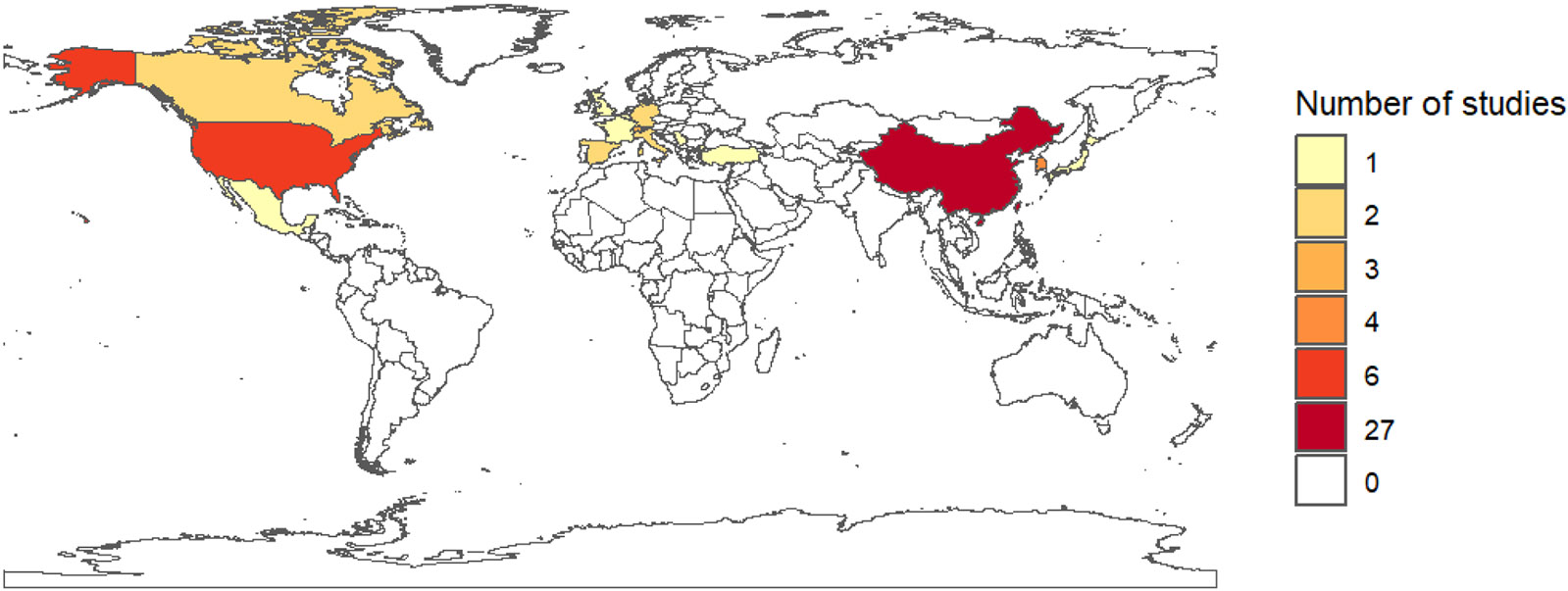
Distribution of studies by countries. Most of the studies were conducted in China (50.09%) and the USA (10.09%), with the rest distributed among various countries in North America, Europe, and East Asia (29.18%).

Out of the 56 studies included in the review, 19 of them focused on making forecasts with or without forecasted data, 15 developed models for interpolation and/or extrapolation, 21 studies were regular predictions at monitoring stations, and one study worked on a downscaling problem. The studies were done in 16 countries around the world. Twenty-eight of them were done in China (50.09%), 6 in the USA (10.09%), 16 in other countries in Nouth America, Europe, and Asia (20.09%), and 5 were done in multiple countries from North America, Europe, and East Asia (9.09%). However, there is a notable research gap in some regions, including South America, Africa and the MENA region. The detailed distribution of the countries is shown in Fig. 2. The studies were published between 2018 and February 2023, with most of them being published in 2022 (see Supplemental Material, Fig. S1). The most studied pollutants were PM_2.5_, O_3_ and NO_2_, with PM_2.5_ being studied in 26 studies, O_3_ in 15 studies, and NO_2_ in 7 studies. The characteristics of the included studies and the interpretation results are provided in Supplementary Material, Tables S3 and S4.

### 3.3 Interpretable Machine Learning

Machine learning models are categorized as black box or white box. White box models are transparent and understandable but often have lower accuracy (Molnar, 2022). In contrast, black box models, while more accurate, lack transparency and require explanation methods (Molnar, 2022).

**Fig. 3.**
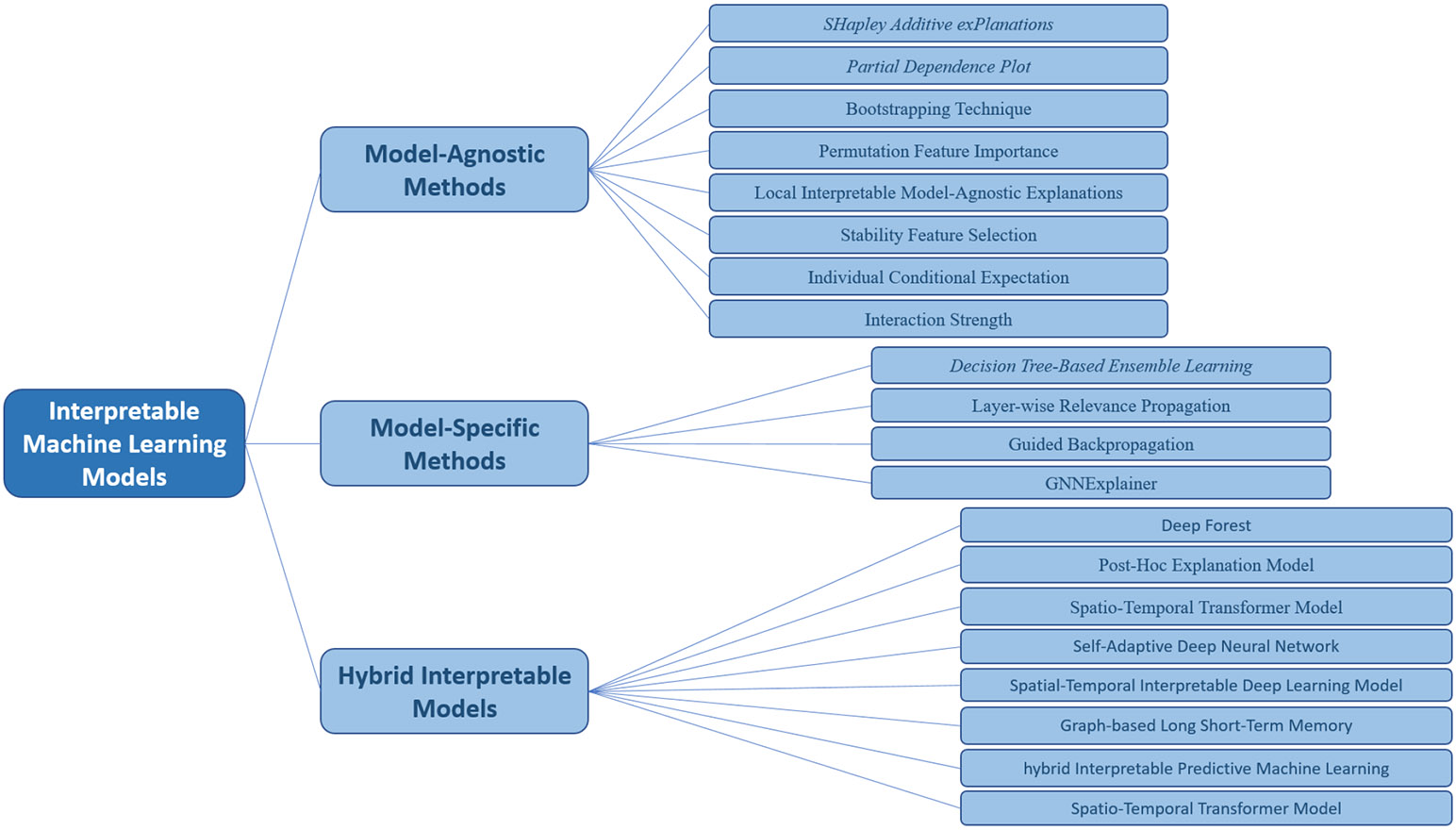
Types of interpretable machine learning models used in the included studies.

Interpretable machine learning encompasses models and methods that make machine learning behavior and predictions understandable (Molnar, 2022). These can be classified as model-agnostic or model-specific. Model-agnostic methods analyze input-output pairs and can be applied to any trained machine learning model (Ribeiro et al., 2016a), while model-specific methods are limited to specific model classes (see Fig. 3).

**Fig. 4.**
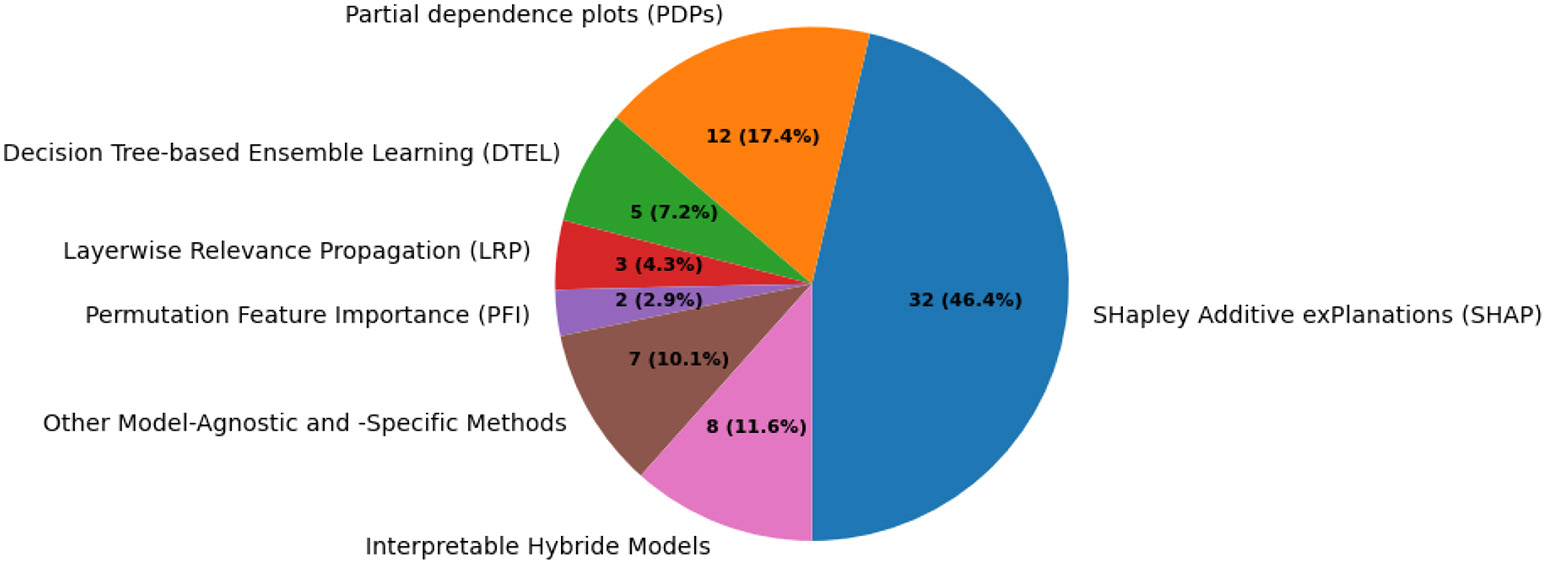
The distribution of the interpretable machine learning models used in the included studies.

In our review, we identified 20 methods: 8 model-agnostic methods, 4 model-specific methods, and 8 hybrid models. The most commonly used method for interpreting air pollution were shapley additive explanations used in 46.4% of the studies, followed by partial dependence plots in 17.4% of the studies, both of which are model-agnostic methods. See Fig. 3 for the types and Fig. 4 for the distribution of other interpretable machine learning models.

#### 3.3.1 Model-agnostic methods

Model-agnostic methods can be classified into global and local methods. Global methods describe the average behavior of a machine learning model, while local methods explain individual predictions to show why the model makes a particular prediction for a specific instance or set of instances (Molnar, 2022). Since global interpretation methods describe average behavior, they are useful for understanding the general mechanisms in the data or debugging a model. Partial dependence plots (PDPs) and shapley additive explanations (SHAP) are frequently used as global and local model-agnostic methods, respectively, to explain air pollution predictions.

##### Partial Dependence Plot (PDP)

The partial dependence plot (PDP), also referred to as a PD plot, is a global, model-agnostic method that illustrates the marginal effect that one or two features have on the predicted outcome of a machine learning model (Friedman, 2001). A PDP can reveal whether the relationship between the target and a feature is linear, monotonic, or more complex. Grange et al. (2018), Li and Sun (2021), Liu et al. (2021), Ren et al. (2020), and Yang et al. (2022a) utilized the PDP to aid in interpretability. Li and Sun (2021) used the PDP to demonstrate the influence of a change in the value of an independent variable on the variation of CO_2_ emissions while holding all other variables constant. Yang et al. (2022a) also used the PDP to visualize the complex relationship between satellite-based aerosol products and PM_2.5_ concentrations. In another study, the PDP was used by Grange et al. (2018) to explain the observed trends and relevant physical and chemical processes influencing PM_10_ concentrations.

##### SHapley Additive explanations (SHAP)

To understand how a machine learning model makes predictions, one approach is to use the shapley value (1953) (Shapley, 1953), which is a cooperative game theory method that can show how fairly the prediction is split among the input features. However, computing the shapley value can be challenging. To make it easier to understand model predictions, Lundberg and Lee (2017) introduced SHAP (SHapley Additive exPlanations), a local method that approximates the contribution of each feature to the prediction using a linear regression model. One variant of SHAP is Kernel SHAP, which trains a linear regression model on samples of the input features to estimate each feature's contribution to the prediction.

There are several methods for visualizing and understanding the contribution of each feature to a prediction. Gu et al. (2021), Song et al. (2022), and Wu et al. (2022) have used SHAP explanation force plots to evaluate the significance of features for the prediction of NO_2_, PM_10_, and PM_2.5_ respectively. In these plots, each Shapley value is a force that either increases (positive value) or decreases (negative value) the prediction. Additionally, the Shapley values can be combined into a global explanation. If we calculate the SHAP values for each instance, we get a matrix with one row for each instance and one column for each feature. We can analyze the entire model by studying this matrix through various methods, such as the SHAP feature importance plot. Choi et al. (2022), Han et al. (2022), Kim et al. (2021), Stadtler et al. (2022), and Zhang et al. (2022) used it to average the absolute Shapley values for each feature across the data. These values are then represented as global importance factors for predicting pollutants like PM_2.5_, NO_2_, O_3_, SO_2_, and PM_10_. Another method is the SHAP summary plot was used to combine feature importance with feature effects (Alvarez and Smith, 2021; Gu et al., 2022; Han et al., 2022; Kang et al., 2021; Kim et al., 2021; Marvin et al., 2022; Nabavi et al., 2021; Ren et al., 2022; Song et al., 2022; Wang et al., 2020; Wei et al., 2022; Wu et al., 2022). This plot can give insights into the relationship between the feature values and their impact on the prediction in each instance. To see the exact form of this relationship, a SHAP dependence plot was used to show the local relationship between the selected features and individual estimates of NO_2_, O_3_, and PM_2.5_ (Alvarez and Smith, 2021; Just et al., 2020; Nabavi et al., 2021).

Force plots, importance plots, dependence plots and also SHAP interaction values were used by Stirnberg et al. (2021) to show the contribution of each feature to the estimate of PM_1.0_, and to highlight the interactions between the features and their impact on PM_1.0_. García and Aznarte (2020) applied all of these methods, including clustering Shapley values, which clusters Shapley values based on their similarity of explanation, to create a plot with several force plots, each explaining the prediction of an instance of NO_2_ concentrations.

##### Permutation Feature Importance (PFI)

The permutation feature importance (PFI) method was used by Ren et al. (2020) and by Shi et al. (2023) to explain the estimation of O_3_ and PM_2.5_ concentrations, respectively, by assessing the significance of individual features in different algorithms. PFI works by shuffling the values of a single feature while keeping the others constant (Fisher et al., 2018) and measuring the resulting drop in model performance. Features that, when permuted, lead to a significant decrease in model performance are considered more important, while those with a minimal impact are less critical. For a more in-depth practical applications, refer to (scikit-learn, 2023).

##### Individual Conditional Expectation (ICE)

Individual Conditional Expectation (ICE) plots are a visualization technique that focuses on understanding how the predictions of a machine learning model change for individual data instances as a specific feature is altered (Goldstein et al., 2015). It was used by Ren et al. (2020) to display the associations between the model prediction of O_3_ and covariate for each sample. ICE plots display one line per instance, showing the relationship between a feature's variation and the corresponding change in prediction. This method is particularly useful for revealing heterogeneity in the relationships between features and predictions, especially in the presence of complex interactions.

ICE plots and partial dependence plots differ in their level of detail. PDPs give a general overview of how a feature impacts predictions across all instances, while ICE plots delve into the individual behavior of each instance (Molnar, 2022). ICE plots help uncover interactions and variations in predictions that may be obscured in PDPs, making them valuable for gaining a deeper understanding of the model's behavior.

##### Bootstrapping technique (BS)

The bootstrapping technique (BS) is a statistical method that estimates the sampling distribution of a statistic by repeatedly resampling from the observed data (James et al., 2013). In the context of machine learning and feature selection, bootstrapping can be used to assess the importance of variables by resampling the dataset and evaluating its impact on model performance. This approach provides a robust means of determining which features have the greatest influence on a model's predictions. Kleinert et al. (2021) employed this technique to evaluate the effects of different input variables on the results of ozone forecasts generated by a deep learning model. They accomplished this by randomly selecting one variable at a time, thereby disrupting its temporal structure in the input data. Subsequently, the model's performance was evaluated using the altered data, and a skill score was calculated to quantify the impact of each variable.

##### Local Interpretable Model-agnostic Explanations (LIME)

Local interpretable model-agnostic explanations (LIME) is a machine learning technique used to explain predictions made by complex machine learning models in a way that is both interpretable and locally faithful (Ribeiro et al., 2016b). Nabavi et al. (2021) used LIME to understand the behavior of their machine learning model for specific instances, illustrating the local relationship between selected features and individual ozone estimations.

LIME starts by selecting a specific instance or data point for which you want to explain the model's prediction. It generates a dataset of perturbed instances by slightly modifying the selected instance. These perturbations may involve making small changes to the features while keeping the label constant. The machine learning model that needs explanation is then used to make predictions on this perturbed dataset. LIME fits an interpretable, locally weighted model (e.g., linear regression) to approximate the behavior of the complex model within the vicinity of the selected instance. This local model should be simple and interpretable. The local model provides an interpretable approximation of how the machine learning model behaves for the specific instance, allowing you to understand the importance of different features and how they contribute to the prediction. The coefficients of the local model can be used to assess the importance of different features for predicting the instance (Molnar, 2022).

##### Stability Feature Selection (SFS)

Stability feature selection (SFS) method was utilized by Meinshausen and Bühlmann (2010) to reduce dimensionality and improve estimator performance. It is a method used when dealing with high-dimensional and limited training data in statistical modeling. Its primary goal is to select the most important features while eliminating noisy ones to enhance model performance and generalization. SFS uses L1 regularization and bootstrapping, repeatedly creating random subsets of data and applying L1 regularization to select features. Features that are consistently chosen across different randomizations are deemed significant. SFS is robust and less sensitive to regularization choices, making it effective for feature selection in challenging modeling scenarios (Meinshausen and Bühlmann, 2010).

##### Interaction Strength (IS)

Interaction strength (IS) or feature interaction (FI) measures the interactions between variables in a statistical or machine learning model. It helps to understand how different variables influence each other and, in turn, affect the model's predictions (Molnar, 2022). Ren et al. (2020) used H-statistics to compute the interaction strength for each variable, explaining O_3_ predictions, with a higher interaction strength value indicating a stronger interaction effect. This can help identify which variable pairs have significant interactions and guide the interpretation of the model's results (Friedman and Popescu, 2008).

#### 3.3.2 Model-specific methods

Model-specific methods are techniques customized to a specific model's needs, addressing its unique challenges and optimizing performance (Molnar, 2022). Model-specific methods may include algorithms, tools, or strategies that are optimized for a particular modeling context.

##### Decision Tree-based Ensemble Learning (DTEL)

Decision tree-based ensemble learning models are a type of machine learning approach that combines multiple decision trees to create a more robust and accurate predictive model. Decision trees (Holzinger, 2015) are simple models that use binary decisions based on input features, and ensemble models utilize several of them to improve predictions.

While decision tree-based ensemble learning models were once considered non-interpretable, they still offer some degree of interpretability by revealing feature importance. Studies have utilized them for predicting air pollution. Extreme gradient boosting (XGBoost) was used by Wang et al. (2022) to evaluate the importance of each variable, and XGBoost and random forest (RF) were used by Coker et al. (2021) to predict fine particulate matter (PM_2.5_) air pollution. Chen et al. (2022) calculated the feature importance while obtaining the distribution of PM_2.5_ based on extra trees (ET). Liu et al. (2021) selected the predictor variables based on gradient boosting decision tree (GBDT) to predict PM_2.5_ and CO concentrations. Finally, Zhai and Chen (2018) generated feature importance scores for forecasting and analyzing PM_2.5_ concentrations using XGBoost and Adaboost, and they visually presented the general structure of tree-based models in the form of a decision tree.

##### Layer-wise Relevance Propagation (LRP)

Layer-wise relevance propagation (LRP) is a technique that can explain complex deep neural networks (Bach et al., 2015). LRP highlights the input features used by the model to make predictions, providing the possibility for the model to explain itself. The basic concept of LRP is to assign relevance scores to each input, showing the contribution of each neuron input to the network output. LRP has been applied to represent the contribution of each input time step in PM_2.5_ forecasting by Kim et al. (2022), to identify informative predictors for PM_2.5_ estimation from horizontal observations by Park et al. (2020), and to visualize and analyze the varying correlation between traffic density and PM_2.5_ concentration in different regions by Du et al. (2023). For more information on LRP, refer to the works of Bach et al. (2015) and Montavon et al. (2019).

##### Guided Backpropagation (GB)

Guided backpropagation (GB) is a technique used in deep learning and neural network interpretability to highlight the importance of different input features or regions for a particular model's prediction (Springenberg et al., 2014). It is often used in the context of convolutional neural networks (CNNs) for image analysis and interpretation. The idea behind Guided Backpropagation is to modify the standard backpropagation algorithm used for training neural networks to only allow positive gradients to flow backward while setting negative gradients to zero. This means that during the backpropagation process, only the paths that increase the activation of a particular feature or neuron are considered, and paths that reduce the activation are effectively ignored.

Steininger et al. (2020) used guided backpropagation to visualize the regions of an input image that a neural network is focusing on for NO_2_ estimation, allowing visualization of which parts of the image the model is paying attention to. This helps in understanding what aspects of the input data have the most influence on the network's decision.

##### GNNExplainer

GNNExplainer is a technique used for explaining the predictions and behaviors of graph neural networks (GNNs). Graph neural networks are a class of deep learning models designed for graph-structured data, and they have gained significant popularity in various domains, including social network analysis, recommendation systems, and bioinformatics (Zhou et al., 2020).

GNNExplainer offers local explanations, revealing why specific nodes are classified as they are and how important edges contribute to decisions Ying et al. (2019). GNNExplainer was utilized by Zhou et al. (2022) to identify critical connections in the graph by extracting the most influential and important subgraph structures for PM_2.5_ prediction at each station, thus enhancing transparency. GNNExplainer often includes visualizations highlighting influential graph elements, aiding intuitive understanding. This tool aligns with broader efforts to make complex models interpretable and supports researchers, practitioners, and domain experts in understanding GNN predictions, diagnosing issues, and enhancing trustworthiness in GNN applications.

#### 3.3.3 Hybrid interpretable models

Some studies have developed new interpretable models by incorporating an element of explainability into their structure to make them more interpretable. For example, Chen et al. (2021) employed a new machine learning model called deep forest (DF), which combines deep neural networks with tree models. They utilized the feature importance derived from the deep forest model to assess the role of variables in predicting near-ground PM_10_ concentration. Bonet et al. (2022) introduced a post-hoc explanation model called NodeSel, which can be used to identify the most closely related nodes to a specific target node by applying both the graph structure and air pollution information. This method provides insights into the connectivity of the graph and its structure. A self-adaptive deep neural network (SADNN) was developed by (Chen et al., 2021) based on the standard deep neural network (DNN). They integrated an attention module into the standard DNN to allow the SADNN model to self-adaptively calibrate the relationships between predictors and PM_2.5_ and provide insight into predictor importance. The advantage of the SADNN model is that it determines the daily significance of predictors for each grid, which differs from decision tree-based ensemble learning models that give overall predictor importance. Yan et al. (2021) developed a spatial-temporal interpretable deep learning model (SIDLM) to predict PM_2.5_ concentrations, and Zang et al. (2021) created a tree-based ensemble deep learning model (semi-SIDLM) to predict O_3_ concentrations. Both models added a linear regression component into their structure to assess the monthly contribution to atmospheric PM_2.5_ and O_3_ levels. Gao and Li (2021) proposed a graph-based long short-term memory (GLSTM) model to predict PM_2.5_ concentration by introducing an adjacency matrix into the long short-term memory (LSTM) cell. They treated all air quality monitoring stations as nodes in a graph and constructed a parameterized adjacency matrix based on the connections between these nodes. By examining the obtained parameter values of the adjacency matrix, they determined the importance of the relationship between the different stations to enhance the interpretability of the model. A new hybrid interpretable predictive machine learning (HIP-ML) model was proposed by Gu et al. (2022) for PM_2.5_ forecasting. The HIP-ML structure is constructed with a deep neural network and a non-linear auto regressive moving average with exogenous input model. By incorporating the OFR algorithm into the HIP-ML model as a feature selection stage, they can determine the significance of each feature that the model considers, at specific times. Finally, the spatio-temporal transformer model (STTM), developed by Yu et al. (2023), is designed for accurate air quality forecasting at monitoring stations. It explaines the factors influencing changes in PM_2.5_ concentration predictions and showcase the model's capability in mapping PM_2.5_ concentrations resulting from wildfire smoke. STTM enhances interpretability by using a hybrid approach that combines spatial, temporal, and value embeddings, along with a sparse attention mechanism. This method considers timestamp order, station locations, and cross-correlation functions to better understand air quality trends and identify hotspots.

## 4 DISCUSSION

The aim of this review was to identify interpretable machine learning models and explanatory methods with the goal of improving model interpretability while maintaining high accuracy for predicting air pollution. The study identified a total of 20 methods. Out of these, 8 were model-agnostic methods, 4 were model-specific methods, and 8 were hybrid models with an element of interpretability incorporated into them. The introduction of each method and its application in air pollution has been presented in the preceding section. In the following sections, we will outline their respective advantages and disadvantages, which are summarized in Table 1.

### 4.1 SHapley Additive explanations (SHAP)

The most commonly used model-agnostic method for interpreting air pollution prediction in the studies included in this review was shapley additive explanations. This method may be the only legally compatible method among the local explanatory methods because it has a solid theoretical basis in game theory (Molnar, 2022). This method fairly distributes the prediction among the feature values (Lundberg and Lee, 2017). In addition to Kernel SHAP, there are other forms of SHAP presented by Lundberg and Lee (2017), such as Tree SHAP or Deep SHAP, which are used for tree-based models and deep neural networks, respectively. However, Kernel SHAP is the only one that is universal and can be applied to any type of machine learning model.

A limitation of the Shapley value is that if features are dependent or correlated, it can assign excessive weight to unlikely data points (Molnar, 2022). The Shapley value of a feature can be prone to misinterpretation. It does not represent the difference in predicted values when removing the feature from the model's training. Rather, the Shapley value indicates the contribution of the feature value to the difference between the actual prediction and the mean prediction, given the current set of feature values (Molnar, 2022). Another challenge is that SHAP can allow the creation of intentionally misleading explanations, potentially concealing biases (Slack et al., 2020). While this may not concern the data scientist generating the explanations, recipients of SHAP interpretations may question their credibility (Molnar, 2022).

### 4.2 Partial Dependence Plot (PDP)

Partial dependence plots (PDPs) are ideal for representing how a feature affects prediction on average, as long as the feature being calculated is not correlated with other features (Molnar, 2022). When features are uncorrelated, a PDP displays how the average prediction in a dataset change as a particular feature changes. The maximum number of features that can be included in a PDP is two, as it is difficult for humans to imagine a world with more than three dimensions. Additionally, the distribution of the features in the PDP is not displayed, which may result in over-interpretation of regions with little to no data (Molnar, 2022). This issue can be easily solved by showing indicators of the data points on the x-axis or by adding a histogram to the plot.

### 4.3 Layer-wise Relevance Propagation (LRP)

LRP can be used to explain the predictions of complex modern neural networks in terms of input features by propagating the prediction backwards through the network. LRP can be applied to various neural network model architectures, including inputs such as images (Bach et al., 2015), videos (Kim et al., 2022), time series (Anders et al., 2019), or text (Arras et al., 2017). This makes it applicable to a large number of practical scenarios that require an explanation of air quality prediction, such as using satellite images or time series observations that represent the movement and transport of atmospheric compositions in the air.

### 4.4 Permutation Feature Importance (PFI)

The feature importance quantifies how much a model's error increases when a feature is removed, providing insight into each feature's impact on predictions. It's a tool applicable to various algorithms (scikit-learn, 2023). PFI automatically takes into account interactions with other features, which can result in both the main feature's effect and interaction effects being included in importance measurements (Molnar, 2022). Notably, it avoids model retraining, saving time when assessing feature importance within a fixed model context, thus preventing variations and misleading conclusions (Molnar, 2022).

Permutation feature importance (PFI) has certain disadvantages. The choice of evaluation metric affects PFI results, as it's linked to the model's error (Molnar, 2022). Therefore, using different metrics may yield distinct rankings of feature importance. PFI introduces randomness through shuffling, and results may vary when repeated (Molnar, 2022). Features that may seem insignificant in a low accuracy model can be significant in a high accuracy model. Therefore, it's essential to understand the model's effectiveness before assessing feature importance (scikit-learn, 2023). Furthermore, when a feature is permuted and correlated with another in the dataset, the model can still access the permuted one through its correlated feature. This situation may lead to low importance scores for both features, even if they are actually important (scikit-learn, 2023). One approach to address this is by clustering the correlated features and retaining one feature from each cluster.

### 4.5 Individual Conditional Expectation (ICE)

Individual Conditional Expectation plots are highly intuitive, as they display individual predictions for each instance when a specific feature is varied. They are particularly effective at revealing heterogeneous relationships that may be concealed by partial dependence plots (Molnar, 2022).

ICE plots have limitations. They are best suited for visualizing one feature at a time, as representing two features would create complex overlays and make the plot difficult to interpret. Additionally, like partial dependence plots, ICE plots can be affected by feature correlations, potentially leading to invalid data points. Drawing many ICE curves can clutter the plot, making it challenging to interpret. To address this, transparency or plotting a sample of lines can be applied. ICE plots may not readily show the average, but this can be resolved by complementing ICE with partial dependence plots for a more comprehensive view (Molnar, 2022).

### 4.6 Bootstrapping technique (BS)

Bootstrapping technique assesses the impact of input variables without requiring model retraining, using the same model weights learned from the full dataset for the evaluation (Kleinert et al., 2021). Preserving the input variable distribution in bootstrapping helps prevent adverse effects when correlated variables are present. However, Kleinert et al. (2021) pointed out that this method may underestimate the impact of a specific variable in the case of correlated input data, as the model tends to focus on the dominant feature, as observed with ozone in their study. It's important to note that this analysis solely evaluates the behavior of their deep learning model and does not assess the real-world impact of these variables on actual ozone formation in the atmosphere.

### 4.7 Local Interpretable Model-agnostic Explanations (LIME)

The key advantage of local interpretable model-agnostic explanations is its ability to explain machine learning model predictions using simple and interpretable surrogate models, even when the underlying model is complex. This interpretability remains consistent if you switch the underlying model (Ribeiro et al., 2016b). LIME provides concise and user-friendly explanations, making it suitable for scenarios where laypersons need to understand model decisions (Ribeiro et al., 2016b).

However, defining the appropriate scope of data instances for explanation purposes remains a challenge, making it difficult to ensure reliable explanations (Molnar, 2022). LIME use of a Gaussian distribution for sampling to create perturbed instances. This can neglect feature correlations, resulting in potentially unrealistic data points that affect the quality and validity of local explanations (Molnar, 2022). Explanations are often unstable, varying between similar instances, making trust an issue (Alvarez-Melis and Jaakkola, 2018). Additionally, LIME explanations can be manipulated, raising concerns about their trustworthiness and potential biases. This underscores the importance of using LIME in machine learning interpretability with caution and critical evaluation (Slack et al., 2020).

### 4.8 Stability Feature Selection (SFS)

Stability feature selection offers robust feature selection, even in non-ideal conditions. It handles high-dimensional data effectively and enhances model generalization, reducing overfitting. SFS simplifies interpretation by focusing on essential features. It's also less sensitive to regularization choices, ensuring ease of implementation (Meinshausen and Bühlmann, 2010).

SFS drawbacks include computational complexity with large datasets, potential information loss due to the exclusion of valuable features, data quality dependency, and limited noise handling, making it less effective when noise follows a pattern.

### 4.9 Interaction Strength (IS)

The H-statistic for interaction strength measures interactions effectively based on strong theoretical foundation. It quantifies variance explained and can identify complex higher-order interactions (Molnar, 2022).

While informative, the interaction H-statistic has some limitations. It's computationally expensive, and its results can be unstable due to sampling when we do not use all data points. Determining the significance of an interaction is challenging since a statistical test for this purpose is not yet available in a model-agnostic version. In cases where features are correlated, the statistic may produce misleading results (Molnar, 2022). It's most useful when not dealing with pixel inputs, and the assumption of feature independence is crucial for accurate results.

### 4.10 Decision Tree-based Ensemble Learning (DTEL)

Decision tree-based ensemble learning models, previously considered non-interpretable methods, still offer a degree of interpretability by providing insights into feature importance.

However, the presence of correlated features in the tree-based may result in a decrease in significance, which may hide some important features that impact prediction. Moreover, they can be sensitive to noise in the data, which can lead to instability in feature importance rankings (Molnar, 2022). Additionally, while interpreting a decision tree-based ensemble model offers a means to show the average importance of each feature across different samples, the visualization of its structure becomes more complicated due to the use of multiple trees (Natekin and Knoll, 2013).

### 4.11 Guided Backpropagation (GB)

Guided backpropagation provides a visual and interpretable way to understand why a neural network made a particular prediction, especially in image classification tasks. It effectively highlights regions of input data that are most relevant to the network's decision, which can be crucial for understanding the model's behavior (Springenberg et al., 2014). However, GB can suffer from gradient saturation, where gradients become very small in deep layers, making it difficult to highlight features effectively. In some cases, GB may not provide a complete understanding of a network's decision, as it can be limited by the complexity of the network architecture.

### 4.12 GNNExplainer

GNNExplainer enhances Graph Neural Network (GNN) interpretability by offering local explanations for node classifications and their dependence on neighboring elements (Ying et al. 2019). It reveals critical graph connections, highlighting essential data relationships, and includes visualizations to aid in understanding influential components.

However, GNNExplainer can introduce computational complexity, especially for large and complex graphs, potentially slowing down the overall analysis process. It's worth noting that GNNExplainer is primarily designed for GNNs, and its applicability to other machine learning models is limited.

### 4.13 Limitations of the Evidence Included in the Review

The overall judgment of the studies included in this review is based on the checklist for maximizing the reproducibility of ecological niche models. All of the articles were rated as high quality, except for three which were rated as medium quality. The medium-rated articles were a result of their lack of inclusion of essential elements concerning the characteristics of the data, such as a comprehensive data description. While the majority of articles being rated as high quality, many of them did not mention the version or the software of the algorithm used. Additionally, a significant number of studies failed to report whether there were any missing values, outliers, errors, or spatial uncertainties in the data, and the methods for addressing these issues.

Most of the methods have been applied to explain the current predictions using the current input, with only a few focusing on explaining predictions for future air pollution. One such method is LRP, which assigns scores to inputs in sequential neural network models (e.g., RNN, LSTM, GRU) and helps identify impactful features from past time periods for forecasting air pollutants. Kernel SHAP has limitations in assigning feature importance in air pollution forecasting (Marvin et al., 2022; Nabavi et al., 2021). However, Deep SHAP (García and Aznarte, 2020; Song et al., 2022) is utilized to assign importance to inputs in sequential neural network models for air pollution forecasting. GNNExplainer Zhou et al. (2022) is used to explain forecasting but is applicable only to GNN models. Other methods, like Bootstrapping (Kleinert et al., 2021), the introduction of adjacency matrix, and OFR algorithm in hybrid models (Gao and Li, 2021; Gu et al., 2022), uncover feature effects in air pollution forecasting. They illustrate the relationship between monitoring stations and feature importance at specific times.

**Fig. 5.**
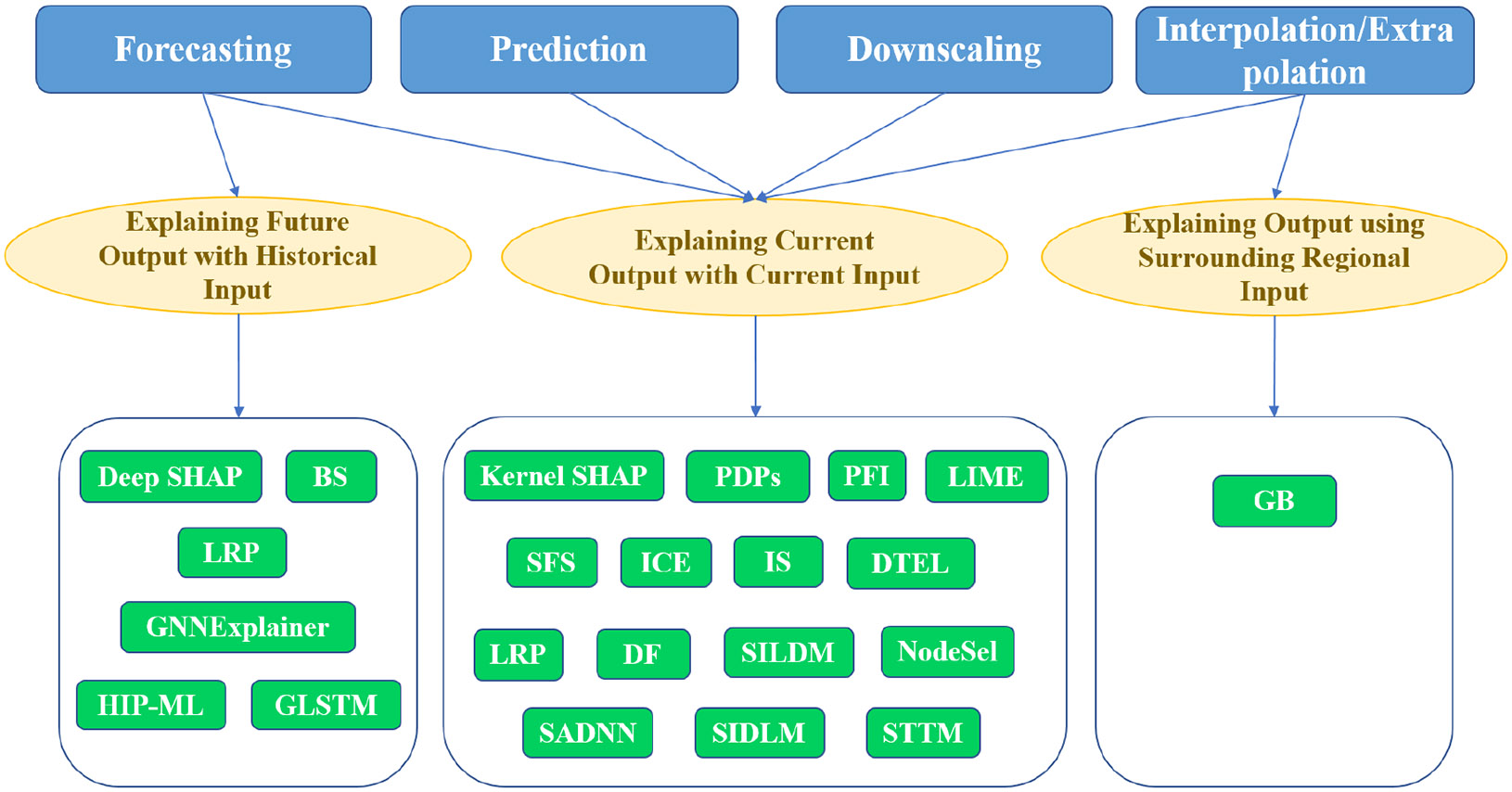
Interpretable machine learning methods for air pollution prediction.

Only Steininger et al. (2020) employed satellite images for air pollution prediction. They used guided backpropagation (GB) as a model-specific method and found that the model prioritizes motorways, trunk roads, and primary roads in map images to predict NO_2_ concentration. This method can be extended to analyze different satellite images from various instruments, identifying regions impacting air pollution in specific areas. For details on the methods used for each objective task, please refer to Fig. 5.

### 4.14 Strengths and Limitations of this Review

This systematic review is the first to discuss interpretable machine learning models aimed at enhancing model interpretability while maintaining high accuracy in predicting air pollution. It serves as a valuable reference for policy-makers, scientists, and researchers in identifying factors contributing to air pollution and developing effective interventions for improved public health. However, our review has limitations: excluding gray literature increases publication bias risk, limited inclusion of studies from five databases may miss relevant research, and the focus is solely on air pollution prediction. Expanding the review to include other application areas would identify additional relevant tools.

### 4.15 Implications of the Results and Future Research

Interpretable machine learning in atmospheric science offers a valuable bridge between data-driven insights and informed decision-making. These techniques identify the most significant features, such as temperature, humidity, and wind speed, used by models for predictions. This not only assist researchers in gaining insights into the crucial atmospheric variables impacting air pollution but also influences outcomes like weather patterns or climate.

Machine learning models often acquire biases from training data, leading to potential discrimination against specific sample groups. Interpretability helps identify and address bias in models. For instance, a pollution-detection model may unfairly target regions already burdened with high pollution levels. This bias could stem from shifting emission-prediction relationships or similar weather conditions to low-pollution areas. The goal is to predict pollution peaks specifically in areas where genuine increases occur. Interpretability helps uncover the root causes of predictions, elucidating the crucial atmospheric variables that influence pollution levels. In the context of air quality management, this can assist policymakers in identifying specific measures to target pollution sources effectively. For example, if the data reveals that certain weather conditions or emissions patterns contribute to pollution peaks, policies can be tailored to address these issues. Moreover, when interpretability methods expose model biases or inequities in pollution predictions, policymakers can take corrective actions to ensure that policies do not unfairly impact certain communities or regions.

Moreover, these techniques can also aid in scenario analysis and forecasting. Policymakers can use these models to simulate the potential outcomes of different policy interventions or climate scenarios. For instance, they can assess the impact of emissions reduction strategies on future air quality or examine the consequences of various climate change mitigation efforts. This proactive approach empowers policymakers to develop evidence-based policies and adapt to changing environmental conditions.

Furthermore, the transparency provided by interpretable machine learning methods fosters effective communication with non-expert stakeholders, including policymakers. The visual and easily digestible explanations generated by these models play a crucial role in translating complex scientific findings into actionable information for decision-makers. This facilitates informed discussions and collaborative efforts between researchers, policymakers, and the public.

Our review highlights research gaps, including the need to utilize explanatory methods on satellite images for examining regional impacts on air pollution predictions. The utilization of explanatory methods in air pollution forecasting is limited, requiring the development of new tools and methods for time series forecasting that can be applied to all types of models. Research on challenges and approaches to mitigate uncertainty associated with these methods is recommended. Additionally, conducting systematic reviews in other application areas to identify new interpretation tools would be valuable.

## 5 CONCLUSION

In this paper, we reviewed interpretable machine learning for air pollution prediction, with shapley additive explanations and partial dependence plots being prominent techniques. These methods identify key features influencing air pollution predictions, thereby enhancing understanding of interactions between atmospheric variables. Ultimately, these approaches can facilitate a better understanding of the complex relationship between air pollution and environmental health, climate change, and urban planning. Policymakers, scientists, and researchers can utilize this resource to gain insights into the crucial atmospheric variables that impact air pollution. Interpretable machine learning serves as a bridge connecting scientific insights and actionable policies, empowering decision-makers to translate data-driven findings into concrete actions.

## Supplementary Material

jj

## Figures and Tables

**Table 1.** Advantages and disadvantages of interpretable machine learning techniques.

Methods	Advantages	Disadvantages
SHapley Additive exPlanations (SHAP)	This method has a solid theoretical basis and evenly distributes predictions among the feature values.	Shapley value can assign excessive weight in dependent features. SHAP may enable misleading explanations, hiding biases. Shapley value isn't difference in predicted values. It reflects feature's contribution to prediction differences.
Partial Dependence Plot (PDP)	PDPs show how one or two uncorrelated features influence predictions.	PDPs often involve up to two features due to human visualization limits. Missing feature distribution can cause over-interpretation in PDP.
Layer-wise Relevance Propagation (LRP)	LRP explains complex neural network predictions with input features, adaptable to various model architectures with diverse data sources.	Sensitivity to network architecture and hyperparameters.
Permutation Feature Importance (PFI)	PFI gauges impact on model predictions. It considers interactions, measuring main and interaction effects in importance.	PFI results vary with evaluation metric choice. PFI's randomness may lead to varying results. Feature significance depends on model accuracy level. Correlation may lower feature importance when permuting one.
Individual Conditional Expectation (ICE)	ICE plots are intuitive, revealing hidden relationships.	ICE plots suit one feature; two can be complex. ICE plots may be influenced by feature correlations. ICE plots may not display averages; pair with partial dependence for completeness.
Bootstrapping technique (BS)	Assesses input variables without model retraining; uses learned model weights.	It may underestimate variable impact with correlated input data.
Local Interpretable Model-agnostic Explanations (LIME)	LIME explains complex model predictions with interpretable surrogate models. LIME offers user-friendly, layperson-accessible explanations.	Challenging scope definition of data instances impacts explanation reliability. Explanations differ among similar instances, raising trust issues. Manipulation raises trust and bias concerns in LIME explanations.
Stability Feature Selection (SFS)	SFS improves robustness, handles high dimensions, and enhances generalization. It simplifies interpretation by focusing on essential features.	SFS has limitations: complexity with large data, potential information loss, data quality dependency, and limited noise handling.
Interaction Strength (IS)	H-statistic measures interactions well with strong theoretical foundation. Quantifies explained variance; identifies complex higher-order interactions.	Computationally expensive, unstable results with incomplete data points. Identifying significant interactions is hard without a model-agnostic test. Correlated features can lead to misleading results.
Decision Tree-based Ensemble Learning (DTEL)	Offer interpretability by revealing feature importance.	Correlated features may reduce feature significance, concealing important predictors. Complex structure due to multiple trees in visualization.
Guided Backpropagation (GB)	Aids neural network understanding, highlighting relevant data regions.	Faces gradient saturation in deep layers, hindering feature highlighting. It may not fully explain network decisions due to complex architecture.
GNNExplainer	Improves GNN interpretability with local explanations and graph visualizations.	It may cause computational complexity, slowing down analysis. GNNExplainer primarily suits GNNs, with limited applicability elsewhere.
