# Supplementary material for: Interpretable Machine Learning Approaches for Forecasting and Predicting Air Pollution: A Systematic Review
Source: Aerosol Air Qual Res. Author manuscript; Available in PMC 2026 Jul 2. (PMC13319251; doi:10.4209/aaqr.230151)
Supplement: jj [file NIHMS2044471-supplement-jj.pdf]

Supplementary Material

Interpretable Machine Learning Approaches for Forecasting and Predicting Air Pollution: A Systematic Review

Anass Houdou[1,2*], Imad El Badisy[1,3], Kenza Khomsi[4], Sammila Andrade Abdala[1,2], Fayez Abdulla[5], Houda Najmi[4], Majdouline Obtel[1,6], Lahcen Belyamani[1,7,8], Azeddine Ibrahimi[1,8], Mohamed Khalis[1,2,6,9]

- [1]*Mohammed VI Center for Research & Innovation, Rabat, Morocco*
- [2]*International School of Public Health, Mohammed VI University of Sciences and Health of Morocco*
- [3]*Inserm UMR912 sciences économiques & sociales de la santé & traitement de l'information médicale (SESSTIM), Marseille, France*
- [4]*Directorate General of Meteorology, Casablanca, Morocco*
- [5]*Civil engineering Department, Jordan University of Science and Technology Irbid 22120, Jordan*
- [6]*Laboratory of Biostatistics, Clinical, and Epidemiological Research, & Laboratory of Community Health (Public Health, Preventive Medicine and Hygiene), Department of Public Health, Faculty of Medicine and Pharmacy, Mohammed V University in Rabat, Morocco*
- [7]*Mohammed VI University of Sciences and Health of Morocco*
- [8]*Faculty of Medicine and Pharmacy, Mohammed V University in Rabat, Morocco*
- [9]*Higher Institute of Nursing Professions and Technical Health, Rabat, Morocco*

[*] Corresponding author. E-*mail address*: ahoudou@um6ss.ma

Supplementary Table S1. The following queries applied to identify scientific literature from the databases.

Database and the date the search was done	Search strategy	Filters and Limitations	Identified studies
Scopus<br>04 April 2022	("Machine Learning" OR "Deep Learning" OR "Neural Network" OR "Data mining" OR "forecast*" OR "Predict*" OR "Artificial Intelligence" OR "Model*" OR "Time serie") AND ("Air quality" OR "air pollution" OR "air pollutant" OR "atmosp*" OR "Sulphur dioxide" OR "Nitrogen dioxide" OR "Ozone" OR "Carbon monoxide" OR "Particulate matter" OR "SO2" OR "NO2" OR "O3" OR "CO" OR "PM10" OR "PM2.5") AND ("Interpretable" OR "Explainable" OR "Black Box" OR "Shap" OR "Shapley" OR "LIME")	Search by: Title, Abstract, Keywords; Language: English; Filter by subject area: Environmental Science, Engineering, Computer Science;	1299
Web of Science<br>04 April 2022	AB=(Machine Learning OR Deep Learning OR Neural Network OR Data mining OR forecast* OR Predict* OR Artificial Intelligence OR Model* OR Time serie) AND AB=(air quality OR air pollution OR air pollutant OR atmosp* OR Sulphur dioxide OR Nitrogen dioxide OR Ozone OR Carbon monoxide OR Particulate matter OR SO2 OR NO2 OR O3 OR CO OR PM10 OR PM2.5) AND AB=(Interpretable OR Explainable OR Black Box OR Shap OR Shapley OR LIME)	Search by: Abstract; Language: English; Research Areas: Engineering, Computer Science, Environmental Sciences Ecology	487
PubMed<br>04 April 2022	((machine learning[Title/Abstract]) OR (deep learning[Title/Abstract]) OR (Neural Network[Title/Abstract]) OR (Data mining[Title/Abstract]) OR (forecast*[Title/Abstract]) OR (Predict*[Title/Abstract]) OR (Artificial Intelligence[Title/Abstract]) OR (Model*[Title/Abstract]) OR (Time serie[Title/Abstract])) AND ((Air quality[Title/Abstract]) OR (air pollution[Title/Abstract]) OR (air pollutant[Title/Abstract]) OR (atmosp*[Title/Abstract]) OR (Sulphur dioxide[Title/Abstract]) OR (Nitrogen dioxide[Title/Abstract]) OR (Ozone[Title/Abstract]) OR (Carbon monoxide[Title/Abstract]) OR (Particulate matter[Title/Abstract]) OR (SO2[Title/Abstract]) OR (NO2[Title/Abstract]) OR (O3[Title/Abstract]) OR (CO[Title/Abstract]) OR (PM10[Title/Abstract]) OR (PM2.5[Title/Abstract])) AND ((Interpretable[Title/Abstract]) OR (Explainable[Title/Abstract]) OR (Black Box[Title/Abstract]) OR (Shap[Title/Abstract]) OR (Shapley[Title/Abstract]) OR (LIME[Title/Abstract]))	Search by: Title/Abstract	269
	((Machine Learning) OR (Deep Learning)) AND ((Air quality) OR (air pollution))	-	600
JuSER<br>10 April 2022	machine learning AND air quality	Search by: Any field	20
	deep learning AND air quality		15
	machine learning AND air pollution		6
	deep learning AND air pollution		2
ScienceDirect<br>04 April 2022	(Machine Learning OR Deep Learning OR Neural Network) AND (Air quality OR air pollution OR air pollutant) AND (Interpretable OR Explainable OR Shap)	Search by: Title, Abstract, Keywords	78
	(Machine Learning OR Deep Learning OR Neural Network) AND (Sulphur dioxide OR Nitrogen dioxide) AND (Interpretable OR Explainable OR Shap)		14

Database and the date the search was done	Search strategy	Filters and Limitations	Identified studies
	(Machine Learning OR Deep Learning OR Neural Network) AND (Ozone OR Carbon monoxide OR Particulate matter) AND (Interpretable OR Explainable OR Shap)		48
	(Machine Learning OR Deep Learning OR Neural Network) AND (SO2 OR NO2 OR CO) AND (Interpretable OR Explainable OR Shap)		76
	(Machine Learning OR Deep Learning OR Neural Network) AND (O3 OR PM10 OR PM2.5) AND (Interpretable OR Explainable OR Shap)		34
	(Data mining OR forecasting OR Predicting) AND (Air quality OR air pollution OR air pollutant) AND (Interpretable OR Explainable OR Shap)		363
	(Data mining OR forecasting OR Predicting) AND (Sulphur dioxide OR Nitrogen dioxide) AND (Interpretable OR Explainable OR Shap)		77
	(Data mining OR forecasting OR Predicting) AND (Ozone OR Carbon monoxide OR Particulate matter) AND (Interpretable OR Explainable OR Shap)		280
	(Data mining OR forecasting OR Predicting AND (SO2 OR NO2 OR CO) AND (Interpretable OR Explainable OR Shap)		453
	(Data mining OR forecasting OR Predicting) AND (O3 OR PM10 OR PM2.5) AND (Interpretable OR Explainable OR Shap)		113
	(Machine Learning OR Deep Learning) AND (Air quality OR air pollution)		613
PubMed<br>28 February 2023	((machine learning[Title/Abstract]) OR (deep learning[Title/Abstract]) OR (Neural Network[Title/Abstract]) OR (Data mining[Title/Abstract]) OR (forecast*[Title/Abstract]) OR (Predict*[Title/Abstract]) OR (Artificial Intelligence[Title/Abstract]) OR (Model*[Title/Abstract]) OR (Time serie[Title/Abstract])) AND ((Air quality[Title/Abstract]) OR (air pollution[Title/Abstract]) OR (air pollutant[Title/Abstract]) OR (atmosp*[Title/Abstract]) OR (Sulphur dioxide[Title/Abstract]) OR (Nitrogen dioxide[Title/Abstract]) OR (Ozone[Title/Abstract]) OR (Carbon monoxide[Title/Abstract]) OR (Particulate matter[Title/Abstract]) OR (SO2[Title/Abstract]) OR (NO2[Title/Abstract]) OR (O3[Title/Abstract]) OR (CO[Title/Abstract]) OR (PM10[Title/Abstract]) OR (PM2.5[Title/Abstract])) AND ((Interpretable[Title/Abstract]) OR (Explainable[Title/Abstract]) OR (Black Box[Title/Abstract]) OR (Shap[Title/Abstract]) OR (Shapley[Title/Abstract]) OR (LIME[Title/Abstract]))	Search by: Title/Abstract	105
Scopus<br>28 February 2023	("Machine Learning" OR "Deep Learning" OR "Neural Network" OR "Data mining" OR "forecast*" OR "Predict*" OR "Artificial Intelligence" OR "Model*" OR "Time serie") AND ("Air quality" OR "air pollution" OR "air pollutant" OR "atmosp*" OR "Sulphur dioxide" OR "Nitrogen dioxide" OR "Ozone" OR "Carbon monoxide" OR "Particulate matter" OR "SO2" OR "NO2" OR "O3" OR "CO" OR "PM10" OR "PM2.5") AND ("Interpretable" OR "Explainable" OR "Black Box" OR "Shap" OR "Shapley" OR "LIME")	Search by: Title, Abstract, Keywords; Language: English; Filter by subject area: Environmental Science, Engineering, Computer Science; Document type: Article; Source type: Journal.	286
Web of Science	AB=(Machine Learning OR Deep Learning OR Neural Network	Search by: Abstract;	158

Database and the date the search was done	Search strategy	Filters and Limitations	Identified studies
28 February 2023	OR Data mining OR forecast* OR Predict* OR Artificial Intelligence OR Model* OR Time serie) AND AB=(air quality OR air pollution OR air pollutant OR atmosp* OR Sulphur dioxide OR Nitrogen dioxide OR Ozone OR Carbon monoxide OR Particulate matter OR SO2 OR NO2 OR O3 OR CO OR PM10 OR PM2.5) AND AB=(Interpretable OR Explainable OR Black Box OR Shap OR Shapley OR LIME)	Language: English; Research Areas: Engineering, Computer Science, Environmental Sciences Ecology, Meteorology Atlospheric Sciences; Document Type: Article.	

Supplementary Table S2. The ecological check list with the additional components.

Section/ Topic	Item	Checklist Item	Page
**Title and abstract**			
**Title**	Prediction model	Identify the multivariable prediction model.	
	Outcome	Identify the outcome to be predicted.	
**Abstract**	Objectives	Provide a summary of objectives.	
	Sample size	Provide the sample size.	
	Predictors	Provide the predictors.	
	Outcome	Provide the outcome.	
	Statistical analysis	Provide the statistical analysis.	
	Results	Provide the results.	
	Interpretation	Provide the interpretation.	
	Conclusions	Provide the conclusions.	
**Introduction**			
**Background and objectives**	Background	Explain the context for developing multivariable prediction model.	
	Objectives	Explain the rationale for developing multivariable prediction model.	
	References	Include references to existing models.	
**Methods**			
**Metadata**	Source of data	Report the source of data.	
	Query/download date or version of the database	Database query/download date or version of the database must be reported. Or the final dataset (that is, after editing and quality control), with the exception of sensitive information (for example, specific locations of endangered taxa), should be deposited in a data archive.	
	Basis of record	Whenever available; Describes how records were originally collected; Whether data are collected opportunistically, as part of structured surveys, as part of repeated surveys, as part of comprehensive checklists of co-occurring species, by scientists, by citizen scientists and so on.	
	Spatial resolution	Report the spatial resolution used for modelling. If aggregation or disaggregation methods used to align the spatial resolutions of variables (for example, if they came from different data providers) should also be reported.	
	Temporal range	Providing the temporal range covered by the environmental variables.	
**Processing data**	Duplicate coordinates	Report if duplicate species records been removed from the same grid square.	
	Spatial and environmental outliers; error	Report if spatial and/or environmental outliers been excluded. (We plotted all the points on maps and excluded any point falling far outside the proven distribution).	
	Spatial and coordinate uncertainty	Uncertainty is a discrepancy between reality and its representation; Spatial uncertainty should be reported when adequate information is available. (For this study, precise locality coordinates for P. solenopsis were not available, so	

Section/ Topic	Item	Checklist Item	Page
		the district-level occurrence data published by Nagrare et al. (2009) were used (n = 42 records)).	
	Sampling bias	Report whether the issues of sampling bias were addressed, and the ways in which this was done.<br>(To reduce the effects of sampling bias, we spatially filtered the occurrence dataset to ensure that no two localities were within 10 km of one another).	
	Spatial autocorrelation	Report if the autocorrelation in the observation has been calculated.	
	Missing data	Describe how missing data were handled (e.g., complete-case analysis, single imputation, multiple imputation) with details of any imputation method.	
**Model calibration (Data input)**	Modeling domain (Study area)	Determine the geographic domain of interest. Delimitation of the domain requires both ecological and practical justification, such as focusing on areas that have been accessible to a species, and areas that have been sampled.	
	Number of background data	Provide the number of data points (cells).	
	Sampling method for background data	Provide the algorithms used to select these points (Random selection, …).	
	Sample size	Provide the sample size of the study.	
	Outcome	Clearly define the outcome that is predicted by the prediction model.	
	Predictors	Clearly define all predictors used in developing the multivariable prediction model,	
	Variable selection	Variable selection procedures must be reported.	
**Model calibration (Algorithm)**	Name	Report the name of modelling algorithm.	
	Theoretical background	Describe the mathematical setting, algorithm, and/or model.	
	Version of algorithm and software	Provide the version library for the algorithm and the software.	
	Parameterization	The parameter or modelling settings should be reported, including default ones.	
**Model output and evaluation**	Format transformation	If the raw model predictions were transformed, provide the methods of transformation (for example, logistic output format).	
	Evaluation index	Specify all indices or measures used to assess model performance.	
	Threshold for evaluation index	If the evaluation indice require a threshold to convert continuous predicted probabilities into a binominal output. Provide how it was estimated.	
	Dataset used to evaluate models	Specify the method for evaluating the model whether using an independent dataset, or part of the dataset not used in model training.	
	Comparison with baseline	Report whether a comparison has been made between several models.	
**Model transfer (Extrapolation)**	Novelty of projected environments relative to training environments	If the model was transferred across space and/or time; Provide the index used to represents how similar the points in the projected environment are to the reference set of points in the calibrated environment, with respect to the set of predictor variables.	
	Collinearity shift between	Quantification of collinearity shift or any steps towards	

Section/ Topic	Item	Checklist Item	Page
	training and projected environments	correcting for it should be specified.<br>(We compared the correlation matrix of the variables in the training region to the average of the correlation matrices of present and future climate layers in the projected area. The highest absolute change of r was 0.3 for bio4 and bio17, and r increased above the 0.7 threshold for 2 pairs of variables (−0.78 for bio3 and bio4; 0.71 for bio16 and bio17; Supplement 2).	
	Extrapolation strategy	The choice of extrapolation strategy, even the default setting of an algorithm, should be provided.	
**Model interpratation**	Methods for interpretability	Methods for explainability or interpretability (eg, saliency maps) and how they were validated.	
**Results**			
**Data**	Data description	Describe the predictors	
		Report how many duplicate species records been removed from the same grid square.	
		Include the number of rows with missing data.	
**Model performance**	Model performance	Report performance measures for the prediction model	
**Model transfer**	Novelty of the projected environments	And quantify the novelty of the projected environments.	
**Discussion**			
**Limitations**	Limitations	Discuss any limitations of the study (such as nonrepresentative sample, few events per predictor, missing data).	
**Interpretation**	Prediction results	discuss the results with reference to performance in the development data, and any other validation data.	
	Interpretation results	Give an overall interpretation of the results	
	Interpretation from similar studies	Considering objectives, limitations, results from similar studies, and other relevant evidence.	
**Implications**	Practical use	Discuss the potential practical use of the model.	
	Future research	Discuss the implications for future research.	

Supplementary Table S3. Main characteristics of the included studies (56).

Reference	Study area	The pollutants of interest	Numbers of observations	Objective of the study	Type of model	Interpretable models / Explanation methods
(Kleinert et al., 2021)	Germany	O_3	1000911	Forecasting	IntelliO3-ts	Bootstrapping technique (BS)
(Zhai and Chen, 2018)	China	$PM_{2.5}$	Not Reported	Forecasting	SEM	Decision Tree-based Ensemble Learning (DTEL)<br>Stability Feature Selection (SFS)
(Wang et al., 2022)	China	$PM_{2.5}$	149094	Interpolation	Hybrid XGBoost-WD	Decision Tree-based Ensemble Learning (DTEL)
(B. Chen et al., 2022)	China	PM_{10}	595466	Interpolation	DF	Deep Forest (DF)
(Coker et al., 2021)	Uganda	$PM_{1.0}$, $PM_{2.5}$ and PM_{10}	269	Prediction	XgbTree, RF, GAM, KNN, RIDGE, LASSO, PCA	Decision Tree-based Ensemble Learning (DTEL)
(Bin Chen et al., 2022b)	China	$PM_{2.5}$	Not Reported	Prediction	ET	Decision Tree-based Ensemble Learning (DTEL)
(Liu et al., 2021)	China	$PM_{2.5}$ and CO	316	Prediction	GBDT	Partial Dependence Plots (PDPs)<br>Decision Tree-based Ensemble Learning (DTEL)
(Zhou et al., 2022a)	China	$PM_{2.5}$	266450	Forecasting	TGGN	GNNExplainer (GNNE)
(Gao and Li, 2021)	China	$PM_{2.5}$	8780	Forecasting	GLSTM	A Graph-based Long Short-Term Memory (GLSTM)
(Steininger et al., 2020)	UK	NO_2	4500	Interpolation	CNN	Guided Backpropagation (GB)
(Du et al., 2023)	China	$PM_{2.5}$	5969	Forecasting	iDeepAir	Layer-wise Relevance Propagation (LRP)
(Kim et al., 2022a)	Soud Korea	$PM_{2.5}$	Not Reported	Forecasting	RNN	Layer-wise Relevance Propagation (LRP)
(Park et al., 2020)	USA	$PM_{2.5}$	249000	Interpolation	CNN	Layer-wise Relevance Propagation (LRP)
(Zang et al., 2021)	China	O_3	Not Reported	Interpolation	semi-SIDLM	Tree-based ensemble deep learning model (semi-SIDLM)
(Yan et al., 2021a)	China	$PM_{2.5}$	335189	Interpolation	SIDLM	Spatial-Temporal Interpretable Deep Learning Model (SIDLM)
(Gu et al., 2022)	China	$PM_{2.5}$	26000	Forecasting	HIP-ML	Hybrid Interpretable Predictive Machine Learning (HIP-ML)
(Li and Sun, 2021)	China	CO_2	Not Reported	Prediction	XGBoost, GBM, SVM, RF	Partial dependence plots (PDPs)
(N. Yang et al., 2022)	China	$PM_{2.5}$	172272	Interpolation	GBNN	Partial Dependence Plots (PDPs)
(Grange et al., 2018)	Switzerland	PM_{10}	186,400	Prediction	RF	Partial Dependence Plots (PDPs)
(Ren et al., 2020)	USA	O_3	249197	Interpolation/Extrapolation	LR, RIDGE, LASSO, ELASTICNET, PCR, PLSR, KNN,	Partial Dependence Plots (PDPs)<br>Permutation Feature Importance (PFI)

Reference	Study area	The pollutants of interest	Numbers of observations	Objective of the study	Type of model	Interpretable models / Explanation methods
					SVR, BPNN, DNN, RT, RF, XGBOOST	Individual Conditional Expectation (ICE) Interaction Strength (IS)
(Chen et al., 2021a)	China	$PM_{2.5}$	1015029	Interpolation	SADNN	Self-Adaptive Deep Neural Network (SADNN)
(Marvin et al., 2021)	Switzerland, Italia	O_3	738	Forecasting	RIDGE, LASSO, ARIMAX, RF, XGBoost, NGBoost, and LSBoost.	SHapley Additive exPlanations (SHAP) Partial Dependence Plots (PDPs)
(Song et al., 2022)	South Korea, China	PM_{10} and $PM_{2.5}$	Not Reported	Forecasting	MTSTL, seq2seq RNN, seq2seq LSTM, seq2seq GRU, Attention networks	SHapley Additive exPlanations (SHAP)
(Choi et al., 2022a)	Soud Korea	PM_{10}	55749	Forecasting	CNNs-LSTM	SHapley Additive exPlanations (SHAP)
(García and Aznarte, 2020)	Spain	NO_2	15635	Forecasting	LSTM	SHapley Additive exPlanations (SHAP)
(Nabavi et al., 2021)	Germany	O_3	198920	Forecasting	MLR-XGBM	SHapley Additive exPlanations (SHAP) Local Interpretable Model-agnostic Explanations (LIME)
(Alvarez and Smith, 2021a)	Spain	NO_2	3560	Forecasting	XGBoost	SHapley Additive exPlanations (SHAP)
(Han et al., 2022)	China	O_3, Temp	8024	Interpolation	LUR-Kriging-RF	SHapley Additive exPlanations (SHAP) Partial Dependence Plots (PDPs)
(Just et al., 2020)	USA	$PM_{2.5}$	692306	Interpolation	XGBoost	SHapley Additive exPlanations (SHAP)
(Kang et al., 2021)	South Korea, eastern China, and Japan	NO_2 and O_3	Not Reported	Interpolation	SVR, RF, XGBoost, LGBM	SHapley Additive exPlanations (SHAP)
(Kim et al., 2021)	Suisse and Italy	NO_2	Not Reported	Interpolation	XGBoost	SHapley Additive exPlanations (SHAP)
(Stadtler et al., 2022)	Europe, North America, and East Asia	O_3	5500	Extrapolation	NN, RF	SHapley Additive exPlanations (SHAP)
(Gu et al., 2021a)	Canada	NO_2	Not Reported	Prediction	LR, RF	SHapley Additive exPlanations (SHAP)
(Stirnberg et al., 2021)	France	$PM_{1.0}$	1086	Prediction	GBRTs	SHapley Additive exPlanations (SHAP)
(Wang et al., 2020a)	Canada	$PM_{2.5}$ and BC	Not Reported	Prediction	LR, ANN, GB	SHapley Additive exPlanations (SHAP)
(Wu et al., 2022a)	China	$PM_{2.5}$	2507	Prediction	LR, Ridge, Lasso, Elasticnet, DT, KNN, SVR, RF, XGBoost, LSTM	SHapley Additive exPlanations (SHAP)
(N. Wei et al., 2022)	China	CO_2 and NO_x	100598	Prediction	RF, XGBoost, LightGBM, and CatBoost	SHapley Additive exPlanations (SHAP)
(Ren et al., 2022)	USA	O_3	249197	Downscaling	BEML	SHapley Additive exPlanations (SHAP)

Reference	Study area	The pollutants of interest	Numbers of observations	Objective of the study	Type of model	Interpretable models / Explanation methods
(S. Zhang et al., 2022a)	China	SO_2	Not Reported	Interpolation	RBE-DA	SHapley Additive exPlanations (SHAP)<br>Partial Dependence Plots (PDPs)
(Ke et al., 2022)	China	$PM_{2.5}$ and O_3	Not Reported	Forecasting	XGBoost-SMOTE	SHapley Additive exPlanations (SHAP)<br>Partial dependence plots (PDPs)
(Ahmad et al., 2022a)	Mexico	O_3	5541	Prediction	RF, GTB and DNN	SHapley Additive exPlanations (SHAP)
(Cheng et al., 2023)	China	O_3	Not Reported	Prediction	XGBoost, LightGBM, CatBoost and RF	SHapley Additive exPlanations (SHAP)
(Lin et al., 2022)	China	$PM_{2.5}$	Not Reported	Prediction	ST-CCN-PM2.5	SHapley Additive exPlanations (SHAP)
(Bai et al., 2023)	China	CO_2	Not Reported	Prediction	XGBoost	SHapley Additive exPlanations (SHAP)
(Aras and Hanifi Van, 2022)	Turkey	EC and CO_2	Not Reported	Forecasting	RF, ANN, KNN, SVM, Adaboost and GBDT	SHapley Additive exPlanations (SHAP)
(Li et al., 2022)	China	$PM_{2.5}$ and O_3	Not Reported	Forecasting	XGBoost	SHapley Additive exPlanations (SHAP)
(Shi et al., 2023a)	China	$PM_{2.5}$	14443.5	Prediction	GBDT and GTJC	Permutation Feature Importance (PFI)<br>Partial dependence plots (PDPs)
(Sadeghi et al., 2022)	USA	O_3	2020	Prediction	CNN, LR	SHapley Additive exPlanations (SHAP)
(Xie et al., 2023)	Not Reported	CO_2	4589	Prediction	RF	SHapley Additive exPlanations (SHAP)
(Bonet et al., 2022)	Belgium	NO_2 and PM_{10}	Not Reported	Prediction	GNNs	NodeSel post-hoc explanation model (NodeSel)
(Liu et al., 2022a)	China	$PM_{2.5}$	Not Reported	Prediction	ST-CCN-IAQI	SHapley Additive exPlanations (SHAP)
(Z. Zhang et al., 2022)	China	$PM_{2.5}$	Not Reported	Prediction	RF	SHapley Additive exPlanations (SHAP)<br>Partial dependence plots (PDPs)
(Yu et al., 2023a)	USA	$PM_{2.5}$	Not Reported	Forecasting	STTM	The spatio-temporal transformer model (STTM)
(Wang et al., 2023)	China	O_3	Not Reported	Forecasting	EML	SHapley Additive exPlanations (SHAP)
(Y. Yang et al., 2022)	China	$PM_{2.5}$	Not Reported	Prediction	LSTM and GRU	SHapley Additive exPlanations (SHAP)
(Luka Jovanovic et al., 2023)	Serbie	Bn	11368	Forecasting	XGBoost	SHapley Additive exPlanations (SHAP)<br>Partial dependence plots (PDPs)

Abbreviation tables for pollutants and machine learning models are provided in the tables S5 and S6.

Supplementary Table S4. Model performance and interpretation results using interpretable machine learning models for each paper

References	Model performance	Results of interpretation
(Kleinert et al., 2021)	Not Reported	The previous-day ozone concentrations are of major importance, followed by temperature to predict ozone concentrations.
(Zhai and Chen, 2018)	R_2 = 0.90 (%)<br>IA = 0.97 (%)<br>MAE = 14.43 μg m^{-3}<br>MAPE = 27.01 (%)<br>RMSE = 23.69 μg m^{-3}	Nitrogen dioxide (NO2) and carbon monoxide (CO) concentrations measured from the city of Zhangjiakou are taken as the most important elements of pollution factors for forecasting PM2.5 concentrations in Beijing, China.<br>Pollutants found in the cities of Zhangjiakou and Chengde have a stronger impact on air quality in Beijing than other surrounding factors.
(Wang et al., 2020b)	R_2 = 80 – 87 (%)<br>RMSE=11.57-12.83 μg m^{-3}<br>MAE = 8.22 - 8.97 μg m^{-3}<br>K-value = 0.79 - 0.86	The three most important variables in the estimation of the low-frequency coefficients of PM2.5 were temperature at 2 m, day of year and longitude. While that in the high frequency coeffcients of PM2.5 were CO, AOD and NO2.
(B. Chen et al., 2022)	R_2 = 82 – 88 (%)<br>RMSE=18.55-23.12 μg m^{-3}<br>AME=11.54-16.82 μg m^{-3}	AOD has the highest contribution to the importance of features in the PM10 model, followed by boundary layer height, temperature, and relative humidity.
(Coker et al., 2021)	RMSE = 7.65 - 16.85 μg m^{-3}<br>R_2 = 24 – 84 (%)	The most important temporal and spatial predictors of monthly PM2.5 levels were monthly precipitation, percent of the population using solid fuels for cooking, distance to Lake Victoria, and greenspace (NDVI) within a 500 m buffer of air monitors.
(Bin Chen et al., 2022b)	R_2 = 0.85 (%)<br>RMSE = 17.77 μg m^{-3}	The feature importance of the bottom layer AOD is higher than that of the upper and total column AOD.
(Liu et al., 2021)	R_2 = 72.24 - 87.55 (%)<br>RMSE=0.0016-0.1025 μg m^{-3}	The meteorological factors, including the relative humidity, temperature, and background wind speed, were important factors influencing the PM2.5 and CO concentrations and contributed to 49.9 (%) and 43.8 (%) to the PM2.5 and CO concentrations, respectively.
(Zhou et al., 2022a)	MAE = 25.935 μg m^{-3}<br>RMSE = 33.721 μg m^{-3}<br>r = 40 (%)<br>IA = 0.566	On August 1, one of the areas of Jiangsu, Zhejiang and Shanghai was dominated by southeasterly winds, and pollutants were mainly transported to the northwest. On November 23, the dominant wind was from the northwest, and the situation is opposite to August 1.
(Gao and Li, 2021)	RMSE = 3.405 μg m^{-3}<br>MAE = 2.606 μg m^{-3}<br>MAPE = 0.126 μg m^{-3}<br>IA = 0.966	The visualization the parameterized adjacency matrix obtained from the end-to-end PM2.5 prediction shows that Longnan area of Gansu Province in China, has the strongest correlation with the nearest air quality station. And in Lanzhou, the most economically developed area of Gansu Province, it has a strong correlation with most air quality monitoring stations in southern Gansu Province, but has a weak correlation with stations in northern Gansu Province.
(Steininger et al., 2020)	R_2= 67.3 (%)<br>RMSE = 8.002 μg m^{-3}	The guided backpropagation shows that the model is paying special attention to motorways, trunk roads, and primary roads when predicting NO2.
(Du et al., 2023)	RMSE = 25.977 μg m^{-3}<br>MAE = 19.313 μg m^{-3}	The five major governing factors for PM2.5 are: air quality, wind direction, weather conditions, pressure and wind speed. Secondary pollutants like O3, and NO2 and traffic emission are also important precursors of PM2.5 which are also important influence factors. The contribution of emissions from the industry and power sectors to the formation of PM2.5 decreased steadily and continuously. Residential emissions are relatively stable while the contribution of traffic emissions increased independently and smoothly.
(Kim et al., 2022b)	MAE = 8.4 μg m^{-3}<br>MBE = 1.4 μg m^{-3}<br>Accuracy = 66.9 (%)<br>POD = 75.1 (%)	The analysis by time-step suggested that the input variables from the numerical models (CMAQ and WRF) play a decisive role in PM2.5 concentration prediction.

References	Model performance	Results of interpretation
		Among the air quality variables, the PM2.5 and PM10 simulated by the CMAQ, and the observed PM2.5 contributed the most to the prediction of the RNN model.
(Park et al., 2020)	R_2= 84 (%)<br>RMSPE = 2.55 μg m^{-3}<br>MPE = 1.56 μg m^{-3}	The top predictors in the model are the meteorological predictors, which have temporal variations and also the land-use and location-related predictors, which do not have temporal variations but have spatial variations.
(Zang et al., 2021)	R_2= 69 – 71 (%)<br>RMSE=21.88-26.59 μg m^{-3}	The results indicate that May, June, and July had the highest contribution to O3, while November and December had the lowest one. In addition, the O3 of the main urban area in Beijing is mainly affected by the Tongzhou and Fangshan districts.
(Yan et al., 2021b)	RMSE=15.30-16.01 μg m^{-3}<br>R_2= 62 – 70 (%)	PM2.5 was clearly highest in winter, especially over the North China Plain (>60 mg m^{-3}). PM2.5 concentrations were lowest in summer, due to reduced concentrations of pollutants and the promotion of diffusion (typically <35 mg m^{-3}).<br>The frequent rainfall that occurred during the monsoon months (JuneeAugust) washed out PM2.5 in the atmosphere, which improved air quality.<br>The haze that frequently occurs in April and May across Beijing can result in extremely high PM2.5 levels, which may explain the high weights determined by SIDLM for these two months.
(Gu et al., 2022)	R = 82.94 (%)<br>PE = 68.78 (%)<br>nRMSE = 0.0752	For 1-hour-ahead PM2.5 predictions (PM2.5 (t)): PM2.5 (t-1), PM2.5 (t-2), Wind (t-1), Pressure (t-3), Sunlight (t-1), Winter (t-1), Precipitation (t-1), Pressure (t-1) and Wind (t-3) are identified as important features.<br>For 3-hour predictive models: The weather factors such as precipitation, humidity and wind directions are selected as top-ranked nonlinear features, for example, Pressure (t-5), PM2.5 (t-3), Humidity (t-5), PM2.5 (t-4), Wind (t-3), PM2.5 (t-4), Humidity (t-3) and Pressure (t-5).
(Li and Sun, 2021)	R_2= 98 (%)<br>RMSE = 7.52<br>rMAE = 0.7 (%)<br>MAE = 6.09<br>re = 0.8 (%)	PDPs indicated that there exists an S-shaped curve for the relationship between CO2 emissions and economic development in China.
(N. Yang et al., 2022)	R_2= 92 (%)<br>RMSE = 7.89<br>MAE = 5.17<br>RPE = 20.54 (%)	AOD is the most notable feature that has a linear overall trend with the PM2.5 concentrations. The larger the AOD value is, the higher the predicted PM2.5 concentration.
(Grange et al., 2018)	R_2= 54 - 71 %<br>MSE = 26.095 - 173.909	Urban traffic sites demonstrated the greatest decrease of −0.77 μg m3 in PM10 concentrations per year. The models also indicated that across Switzerland, elevated PM10 concentrations occur in poor dispersion conditions as well as at high temperatures with a deep boundary layer due to high rates of secondary PM10 generation resulting from photochemical processes.
(Ren et al., 2020)	RMSE = 5.51 - 7.33<br>R_2= 53 – 71 (%)	CMAQ-estimated ozone, daily maximum temperature, open water coverage, pasture coverage, deciduous forest coverage, latitude, longitude, daily maximum relative humidity, wind speed, stationary emissions of PM10, daily minimum temperature, wind direction, stationary emissions of VOC, stationary emissions of NOx, elevation and population density were the top important variables for ozone estimation.
(Chen et al., 2021b)	R_2= 86 – 84 (%)<br>RMSE=13.07-14.30 μg m^{-3}<br>MAE = 8.23 - 8.82 μg m^{-3}	The boundary layer height, elevation and AOD were the most important predictors both spatially and temporally for PM2.5.

References	Model performance	Results of interpretation
(Marvin et al., 2021)	RMSE = 17.68<br>MAE = 13.46<br>MAPE = 10.34<br>S = 0.430<br>Accuracy = 71.43 (%)	The most important feature on the ozone forecast was a value of forecasted temperature.<br>At high concentrations of NO2, higher temperatures accelerate the O3 formation rate. On the other hand, at low concentrations of NO2, O3 generation from NO2 becomes increasingly important with increasing temperature.<br>A partial dependence plot of the measured O3, that is, the target variable and the measured temperature shows an exponential-like relation.
(Song et al., 2022)	MASE = 0.819 - 0.861<br>RMSE = 8.830 - 14.044<br>MAE = 6.324 - 10.117	The features that contributed to PM10 predictions were: PM10, Shenyang_PM10, Shanghai_CO, Dalian_SO2, O3 etc.<br>A force plot is presented to show the contribution of each feature of an instance in Seoul on January 7, 2020, when the PM2.5 predicted value was 0.21. The features in red are those that contributed to raising PM2.5 prediction to 0.21, for example, air features in China such as CO and PM2.5 in Shenyang, and PM10 in Delian. Meanwhile, features such as SO2 in Qingdao, CO in Shanghai, and O3, indicated in blue, contributed to lowering PM2.5 predicted value to 0.21.<br>The contribution of each feature of an instance in Seoul on January 25, 2020, when the PM2.5 prediction value was 0.11 were showed as well.
(Choi et al., 2022b)	R_2= 94.37 (%)<br>RMSE = 6.6186<br>MAPE = 13.5737<br>MAE = 4.4503	The most influential factor for predicting PM10 in Soud Korea (Gwanak, Namdong, Yuseong, and Sasang) at time t + 1 was the PM10 at time [t - 2, t]. CO was the next most influential factor, but its influence was quite small compared to PM10.<br>For Namdong and Yuseong, NO2 was the next most important factor. Compared to that parameter, O3 was more meaningful contributor in Gwanak and SO2 was more meaningful contributor in Sasang.<br>SO2 made smaller contributions than the other air pollutants except the Sasang region.
(García and Aznarte, 2020)	Not Reported	Wind speed has the greatest impact on the predictions of NO2 in the city of Madrid.<br>In the same way, solar radiation and relative humidity features behave similarly, and also show a significant impact in the prediction value.<br>Temperature, values of NO2 concentrations for one day before and wind direction are directly proportional to NO2 concentrations.
(Nabavi et al., 2021)	RMSE = 9.49 μg m^{-3}	SHAP showed an increasing effect of temperature on O3 concentrations which intensifies for temperatures exceeding 17 °C.<br>According to LIME, O3 concentration peaks are mainly governed by meteorological factors under dry and warm conditions on a regional scale, whereas local nitrogen oxide concentrations control base O3 concentrations during cold and wet periods.
(Alvarez and Smith, 2021b)	MSE = 0.0089<br>RMSE = 0.0943<br>MAE = 0.0763<br>MAPE = 0.2611<br>SMAPE = 0.2666	The basic fnding is that the increase in wind speed may efect on pollution reduction.
(Han et al., 2022)	R_2= 65 – 93 (%)<br>RMSE = 0.92 - 28.18<br>MSE = 0.85 - 794.07<br>MAE = 0.7 - 20.31	Humidity and high temperature have a significant positive effect on O3.<br>Maximum wind speed and average wind speed negatively affect O3 concentration.
(Just et al., 2020)	RMSE = 3.22 - 3.57 μg m^{-3}	In the Northeastern US, a low AOD more consistently contributes to a lower PM2.5 prediction, while higher AOD values correspond to

References	Model performance	Results of interpretation
		higher SHAPs but with a substantially more diffuse point cloud.
(Kang et al., 2021)	R_2= 70 – 78 (%)<br>RMSE = 4.75 - 11.11 ppb<br>nRMSE=19.6- 38.3 (%)	The NO2 vertical column density among the TROPOMI-derived variables showed the largest contribution in both the NO2 and O3 models.
(Kim et al., 2021)	R_2= 59 (%)<br>MAE = 7.69 μg m^{-3}	Local interpretations of the machine learning model demonstrate that TROPOMI NO2 satellite observations make a strong contribution to the information content of the near-surface NO2 maps.
(Stadtler et al., 2022)	R_2= 56.99 - 58.34 (%)<br>RMSE = 3.87 - 4.08 ppb	The largest variables of significance for O3 prediction were absolute latitude, relative altitude, forest in the 25 km area, and altitude.
(Gu et al., 2021b)	RMSE = 2 - 2.5 ppb	Density of the population, total length of major roads, industrial land cover, and density of truck during morning rush hour are the most influential predictor variables in terms of higher sum of magnitudes of SHAP values over all test data points.<br>The high vehicle density dominates positive contributions (pushing the NO2 concentrations up), and low vehicle density dominates negative contributions (pulling the NO2 concentrations down).
(Stirnberg et al., 2021)	R_2= 58 (%)	Model results suggest that winter pollution episodes are often driven by a combination of shallow mixed layer heights (MLHs), low temperatures, low wind speeds, or inflow from northeastern wind directions.
(Wang et al., 2020b)	Not Reported	For BC, the most influential variables are major road length, traffic density, vehicle location and speed, daily traffic (AADT), wind speed, and relative humidity.<br>For PM2.5, the most influential variables are temperature, distance to shore, major road length, vehicle location and speed, daily traffic (AADT), distance to major, wind speed, and relative humidity.
(Wu et al., 2022b)	RMSE = 19.58 μg m^{-3}<br>MAE = 15.11 μg m^{-3}<br>RPD = 1.15<br>LCCC = 0.44	The SHAP analyses revealed that the meteorological factors had diferent infuences in specifc predictions.
(N. Wei et al., 2022)	R_2= 71 – 82 (%)	For CO2, the most influential features are the average velocity in the first ten seconds (Avg.V) and vehicle specific power (VSP). Higher values of these characteristics lead to higher SHAP values, which indicates a greater positive feedback contribution to the transient emission values of CO2.<br>For NOx, the exhaust gas temperature range (TR) is the most important feature of the model. Specifically, larger values of the TR correspond to negative SHAP values, indicating a negative feedback effect on the final NOX emissions.
(Ren et al., 2022)	RMSE = 6.62 - 8.14 ppb<br>R_2= 57 – 75 (%)	CMAQ simulations of ozone had the highest contribution to O3 predictions; however, contributions of certain fine-scale covariates such as temperature and solar radiation are also significant.
(S. Zhang et al., 2022b)	R_2= 89 (%)<br>RMSE = 10.7<br>RPE = 39.3	The high variance associated with the model prediction of SO2 was attributable to the falsely specified interactions between the meteorological and land-use variables.<br>By taking the model prediction of SO2 in 2019, a strong interaction between the surface skin temperature (TS) and the land-use variables, such as the water area was found.
(Ke et al., 2022)	R = 87 – 89 (%)<br>ME = 10.34-14.53 μg m^{-3}<br>RMSE = 16.53-18.83 μg m^{-3}	The SHAP and partial dependence plots reveal that geographic features such as latitude and altitude significantly affect the optimized PM2.5 concentrations with positive correlation, as do features such as PM2.5, PM10, humidity, and HOUR. Physical features such as mixing ratio, precipitable water, temperature, sea level pressure, U and V component wind are negatively correlated

References	Model performance	Results of interpretation
		with optimized PM2.5 concentrations. Temperature significantly affects the optimized O3 concentration with a positive correlation.
(Ahmad et al., 2022b)	R_2= 92 (%)<br>IOA = 97 (%)	The shortwave ultraviolet-A predictor was the most important variable in predicting ozone mixing ratios. Low values of shortwave ultraviolet-A reduced ozone mixing ratios, while high values increased ozone mixing ratios. High levels of NO and NO2 both had positive effects on ozone mixing ratios. Relative humidity had a negative effect on ozone mixing ratios, with high values causing a decrease and low values enhancing ozone mixing ratios. Temperature and wind speed also had effects on ozone mixing ratios, with high temperatures pushing down predictions and high wind speeds decreasing predictions for low values. The height of the planetary boundary layer had a negative effect on ozone mixing ratios, with high values pushing down predictions and low values increasing predictions.
(Cheng et al., 2023)	R_2= 96 (%)	The ozone formation in Shenzhen was more affected by volatile organic compounds, of which vehicle emission sources may have the greatest impact.
(Lin et al., 2022)	MSE = 4.94<br>RMSE = 2.17<br>MAE = 1.31<br>R_2= 92 (%)	Shapley analysis shows wind speed is the most influencing factor in fine-grained PM2.5 concentration prediction. The effects of CO and temperature on PM2.5 prediction are moderately significant.
(Bai et al., 2023)	R_2= 95 (%)	SHapley Additive explanation (SHAP) values calculated for all samples showed that the five features that contributed most to the CO2 concentration were H/W, d (along-canyon position of the sensor), T (air temperature), W/2 (cross-canyon position of the sensor), and b_ws (atmospheric environmental background wind speed).
(Aras and Hanifi Van, 2022)	For CO_2:<br>MAE = 11.513<br>RMSE = 14.062<br>sMAPE = 3.974<br>R_2= 95.4 (%)<br>For EC:<br>MAE = 2865<br>RMSE = 3387<br>sMAPE = 3.755<br>R_2= 93.6 (%)	The results obtained show that the total electricity generation from different energy sources is found to be the most important variable interacting positively with both energy consumption and CO2 emissions.
(Li et al., 2022)	R = 96 - 97 (%)<br>RMSE = 15.87 - 21.08<br>MAE = 11.52 - 12.42	The SHAP value showed that, aerosol ion concentration of Sodium and Nitrate concentration were the important features affecting the prediction of PM2.5 concentration by the XGBoost algorithm, and the most important factor affecting O3 concentration is temperature.
(Shi et al., 2023b)	R_2= 88.98 (%)<br>RMSE = 11.3814 $\mu g\ m^{-3}$<br>MAE = 7.4157 $\mu g\ m^{-3}$	The top-4 important features for estimating PM2.5 concentrations using VI, are the temporal codes (TC), geospatial codes (GC), aerosol optical depth (AOD), and temperature (TEM). The PDPs of GTJC show that the estimated PM2.5 concentrations increased with the rising temperature. The PDPs of the GBDT model suggest that there is an inverse relationship between TEM and PM2.5.
(Sadeghi et al., 2022)	R = 74 – 86 (%)<br>IOA = 85 – 92 (%)<br>MAB = 5.18 - 10.21 ppb<br>RMSE = 6.61 - 13.64 ppb	The specific humidity and temperature contributed the most to ozone formation over the Houston and Dallas metropolitan areas. The solar radiation strongly impacted ozone variation over West Texas during from 2000 to 2019.
(Xie et al., 2023)	Not Reported	The results showed that textural property is more important than chemical composition in affecting CO2 uptake. SHAP indicated that

References	Model performance	Results of interpretation
		high CO_2 adsorption capacity appeared at low pressure, temperature of 298 K with a specific surface area.
(Bonet et al., 2022)	Not Reported	The approach highlighted the importance of nodes that are closest in terms of geodesic distance and in the same or neighboring streets, as well as nodes that are further away but share similar characteristics with the original node to be explained.
(Liu et al., 2022b)	RMSE = 9.873<br>MAE = 7.469<br>R_2= 91.7 (%)	Shapley analysis showed PM10, humidity and NO2 were the most influencing factors to predict PM2.5.
(Z. Zhang et al., 2022)	Not Reported	According to the results, human-caused emissions and meteorological conditions were responsible for approximately 67 (%) (40.5 mg m^{-3}) and 33 (%) (19.7 mg m^{-3}) of the variation in PM2.5 concentrations, respectively. Of all the sources, secondary nitrate (SN) had the largest impact, accounting for around 45 (%) of the variation.
(Yu et al., 2023b)	RMSE = 6.92 μg m^{-3}<br>MAE = 4.0 μg m^{-3}<br>BIASE = 0.51 μg m^{-3}	When there is a wildfire, the attention is focused on the inverse distance weighted sum of brightness temperature from grids within any CAL FIRE fire perimeter at the current hour (IDWFI_FP) and the inverse distance weighted sum of brightness temperature from all grids available at the current hour (IDWFI), which shows hotspots at different past hours. However, when there is no wildfire, the hotspot of attention is on traffic flow during hours 2-5. Notably, the past PM2.5 concentrations may not be the most important variable in this scenario.
(Wang et al., 2023)	R_2= 95 (%)<br>MAE = 4.64 μg m^{-3}<br>RMSE = 7.52 μg m^{-3}	The SHAP model identified meteorological variables (temperature, humidity, solar radiation, visibility, and wind speed) and air quality features (NOx, NO2, CO, TSP, and TVOC) as the main contributors to O3 concentrations. Higher levels of solar radiation, maximum temperature, and minimum relative humidity tended to promote higher O3 concentrations. The model also revealed that reducing NOx and increasing VOCs could increase O3 concentrations.
(Y. Yang et al., 2022)	MAE = 0.4306 μg m^{-3}<br>MSE = 0.5567 μg m^{-3}<br>RMSE = 0.7085 μg m^{-3}<br>R_2= 85.51 (%)	The largest contribution to PM2.5 prediction is atmospheric pressure, followed by humidity and temperature.
(L. Jovanovic et al., 2023)	MSE = 0.933440<br>R_2= 91.37 (%)<br>R = 95.5894 (%)<br>MAE = 0.506238<br>RMSE = 0.966147	Toluene and the finest aerosol particles are the most significant factors that affect benzene concentrations, while other important factors include temperature, soil moisture content, wind direction, and the concentrations of nonmethane hydrocarbons and nitrogen oxides. That toluene and benzene have different reactivity and atmospheric half-life. Temperature is a significant parameter that influences benzene concentrations, with a complex impact that decreases with increasing temperature.

Abbreviations for metrics performance are provided in the table S7.

Supplementary Table S5. List of abbreviation (Machine Learning Models)

Abbreviations	Definitions
IntelliO3-ts	Multiple convolutional neural network
SEM	Stacked Ensemble Model
Hybrid XGBoost-WD	Hybrid eXtreme Gradient Boosting with Wavelet Decomposition
DF	A new machine learning deep forest
XGBTree	eXtreme Gradient Boosting Tree
RF	Random Forest
GAM	Generalized Additive Model
KNN	k-Nearest Neighbors
RIDGE	Ridge regression
LASSO	Least Absolute Shrinkage and Selection Operator
PCA	Principal Component Analysis
ET	Extra Trees
GBDT	Gradient Boosting Decision Tree
IAP	Input-Adaptive Proxy
DT	Decision Tree
SVR	Support Vector Regression
SNN	Shallow Neural Network
LSTM	Long Short-Term Memory
MRA	Multiple Regression Analysis
TGGN	A Theory-Guided Graph Network
GLSTM	A Graph-Based Long Short-Term Memory
CNN	Convolutional Neural Networks
LURK-Vecchia	Land-Use Regression Kriging with Vecchia
iDeepAir	A Novel Deep Learning Network
RNN	Recurrent Neural Network
LR	Linear Regression
SARMA	Seasonal Autoregressive Moving Average
MFR	Meteorological Factors Regression
BPNN	Back-Propagation Neural Network
SILDM	Tree-based Ensemble Deep Learning Model
SIDLM	Spatial-Temporal Interpretable Deep Learning Model
HIP-ML	Hybrid Interpretable Predictive Machine Learning
XGBoost	Extreme Gradient Boosting
GBM	Gradient boosting Model
SVM	Support Vector Machine
GBNN	Gradient Boosting of Multi-Layer Neural Networks
ELASTICNET	Elasticnet Regularization
PCR	Principal Component Regression
PLSR	Partial Least Squares Regression
DNN	Deep Feed-Forward Neural Network
RT	Regression Tree
SADNN	Self-Adaptive Deep Neural Network
ARIMAX	Autoregressive Integrated Moving Average with eXplanatory Variable
NGBoost	Natural Gradient Boosting
LSBoost	Least-Squares Boosting
MTSTL	Multi-task and single-task learning
seq2seq RNN	Sequence to Sequence Recurrent Neural Network
seq2seq LSTM	Sequence to Sequence Long Short-Term Memory
seq2seq GRU	Sequence to Sequence Gated Recurrent Units
Attention networks	Attention networks
MLR-XGBM	Multiple Linear Regression-Based eXtreme Gradient Boosting Machines
LUR-Kriging-RF	integrated prediction model based on random forest algorithm

Abbreviations	Definitions
LGBM	Light Gradient Boosting Machine
NN	Neural Network
GBRTs	Gradient Boosted Regression Trees
ANN	Artificial Neural Networks
GB	Gradient Boost
LightGBM	Light Gradient Boosting Machine
CatBoost	Categorical Boosting
BEML	Flexible Bayesian Ensemble Machine Learning
RBE-DA	Robust Back Extrapolation via Data Augmentation
GTB	Gradient Tree Boosting
ST-CCN-PM2.5	Spatial–Temporal Causal Convolution Network
GTJC	Geospatial-Temporal Joint Codes
GNN	Graph Neural Networks
ST-CCN-IAQI	Spatial–Temporal Causal Convolution Network
STTM	SpatioTemporal Transformer Model
EML	Ensemble Machine Learning
GRU	Gated Recurrent Unit

Supplementary Table S6. List of abbreviation (Air pollutants)

Abbreviations	Definitions
PM10, PM2.5, PM1.0	Particulate Matters
SO2	Sulfur dioxide
O3	Ozone
NO2	Nitrogen dioxide
BC	Black carbon
Bn	Benzene
To	Toluene
Fo	Formaldehyde
Ac	Acrolein
NOx	Nitrogen oxides
UFPs	Ultrafine particles
Pb	Lead
Mn	Manganese
Zn	Zinc
Fe	Iron
OP	Oxidative potential
Si	Silicon
S	Sulfur
K	Potassium
Ti	Titanium
Mn	Manganese
Ni	Nickel
Cu	Copper
Zn	Zinc
CO2	Carbon dioxide
CO	Carbon Monoxide
EC	Energy Consumption

Supplementary Table S7. List of abbreviation (Metrics of Performance)

Abbreviations	Definitions
R2	Coefficient of Determination
IA	The Index of Agreement
bias	The Average Error
MAE	Mean Absolute Error,
MAPE	Mean Absolute Percentage Error
RMSE	Root Mean Squared Error
K-value	The Slope of the Fitting Line
AME	Absolute Mean Error
r	Pearson correlation coefficient
MSE	Mean Squared Error
CRPS	Continuous Ranked Probability Score
MBE	Mean Bias Error
POD	Probability Of Detection
RMSPE	Root Mean Squared Percentage Error
MPE	Mean Percentage Error
adj-R2	Adjusted R-Squared
PE	Prediction Efficiency
NRMSE	Normalized Root Mean Squared Error
RMAE	Relative Absolute Mean Rrror
re	Relative Error
RPE	Relative Prediction Error
S	Forecast Skill
MASE	Mean Absolute Scaled Error
SMAPE	Symmetric Mean Absolute Percentage Error
RPD	Ratio of Performance to Deviation
LCCC	Lin's Concordance Correlation Coefcient
BIASE	Bias Error
IOA	Index Of Agreement
MAB	Mean Absolute Bias
ME	Mean Error

Supplementary Table S8. List of full-text articles excluded and reasons for exclusion (n = 58)

List of full-text articles excluded	Reasons for exclusion
(Zhu et al., 2021)	Did not provide interpretation.
(Wang et al., 2018)	Did not provide interpretation.
(Vettori et al., 2019)	Did not provide interpretation.
(Zaidan et al., 2019)	Did not provide interpretation.
(Zhou et al., 2022b)	Did not provide interpretation.
(De Hoogh et al., 2018)	Did not provide interpretation.
(Awang et al., 2015)	Did not provide interpretation.
(Meng et al., 2018)	Did not provide interpretation.
(Kerckhoffs et al., 2019)	Did not provide interpretation.
(Lovrić et al., 2021)	Did not provide interpretation.
(Cabaneros et al., 2020)	Did not provide interpretation.
(Qi and Hankey, 2021)	Did not provide interpretation.
(Varde et al., 2022)	Did not provide interpretation.
(Luo et al., 2022)	Did not provide interpretation.
(J. J. Y. Zhang et al., 2022)	They used interpretable model but they did not provide interpretation.
(Adams and Kanaroglou, 2016)	No explicit method of interpretation was provided.
(Sun and Archibald, 2021)	They presented some parameters related to the modle intuitively by mapping, so that the whole process of assimilation is traceable and interpretable. But the process how they presented the parameters is not provided.
(Yan et al., 2020)	They used a method to reduce the dimensions of the output model, not for the purpose of interpretation.
(Yin et al., 2022)	Not a method of interpretation.
(Ronquillo-Lomeli et al., 2022)	Not available
(Kleinert et al., 2019)	Not available
(Ning Wei et al., 2022)	Not available
(Sun et al., 2023)	Not available
(Peng et al., 2022)	Not available
(Lucena-Sánchez et al., 2021)	Interpretable models with low accuracy
(Kumar and Goyal, 2011)	Interpretable models with low accuracy
(Xu et al., 2022)	Interpretable models with low accuracy
(Fung et al., 2021)	Interpretable models with low accuracy

(Hu et al., 2021)	Interpretable models with low accuracy
(Lešnik et al., 2019)	Interpretable models with low accuracy
(Messier and Katzfuss, 2021)	Interpretable models with low accuracy
(Wu et al., 2014)	Interpretable models with low accuracy
(Iglesias-Gonzalez et al., 2020)	Interpretable models with low accuracy
(Tularam et al., 2021)	Interpretable models with low accuracy
(Naughton et al., 2018)	Interpretable models with low accuracy
(Jin et al., 2019)	Interpretable models with low accuracy
(Cai et al., 2020)	Interpretable models with low accuracy
(Huang et al., 2017)	Interpretable models with low accuracy
(Gaeta et al., 2016)	Interpretable models with low accuracy
(Masri et al., 2019)	Interpretable models with low accuracy
(Ge et al., 2022)	Interpretable models with low accuracy
(Tripathy et al., 2019)	Interpretable models with low accuracy
(Habermann et al., 2015)	Interpretable models with low accuracy
(Luminati et al., 2021)	Interpretable models with low accuracy
(Shi et al., 2020b)	Interpretable models with low accuracy
(Liu et al., 2015)	Interpretable models with low accuracy
(Wu et al., 2017)	Interpretable models with low accuracy
(Shi et al., 2020a)	Interpretable models with low accuracy
(Hellack et al., 2017)	Interpretable models with low accuracy
(Sanchez et al., 2018)	Interpretable models with low accuracy
(Michanowicz et al., 2016)	Interpretable models with low accuracy
(Ho et al., 2015)	Interpretable models with low accuracy
(Jones et al., 2020)	Interpretable models with low accuracy
(Eeftens et al., 2019)	Interpretable models with low accuracy
(Gryparis et al., 2014)	Interpretable models with low accuracy
(Zhang et al., 2015)	Interpretable models with low accuracy
(Chen et al., 2023)	Interpretable models with low accuracy
(Bin Chen et al., 2022a)	Interpretable models with low accuracy

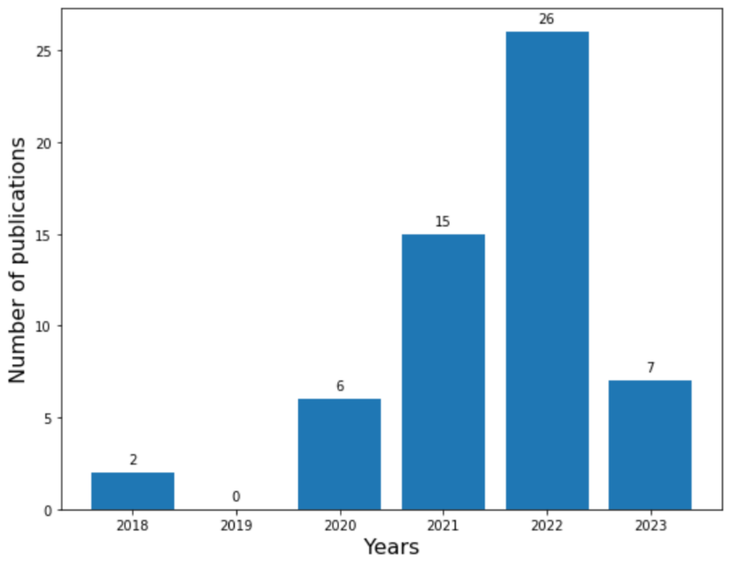

Supplementary Fig. S1. Number of publications per year between January 2011 and February 2023.

References for Supplementary Material

Adams, M.D., Kanaroglou, P.S. (2016). Mapping real-time air pollution health risk for environmental management: Combining mobile and stationary air pollution monitoring with neural network models. J. Environ. Manage. 168, 133–141. https://doi.org/10.1016/j.jenvman.2015.12.012

Ahmad, M., Rappenglück, B., Osibanjo, O.O., Retama, A. (2022a). A machine learning approach to investigate the build-up of surface ozone in Mexico-City. J Clean Prod 379, 134638. https://doi.org/10.1016/j.jclepro.2022.134638

Ahmad, M., Rappengluck, B., Osibanjo, O.O., Retama, A. (2022b). A machine learning approach to investigate the build-up of surface ozone in Mexico-City. JOURNAL OF CLEANER PRODUCTION. https://doi.org/10.1016/j.jclepro.2022.134638

Alvarez, F., Smith, M. (2021a). Using Shapley values to assess the impact of temporary traffic restrictions on NO2 levels in Madrid urban area. Int J Env. Sci Technol 18, 3343–3356. https://doi.org/10.1007/s13762-020-03091-3

Alvarez, F., Smith, M. (2021b). Using Shapley values to assess the impact of temporary traffic restrictions on NO2 levels in Madrid urban area. INTERNATIONAL JOURNAL OF ENVIRONMENTAL SCIENCE AND TECHNOLOGY. https://doi.org/10.1007/s13762-020-03091-3

Aras, S., Hanifi Van, M. (2022). An interpretable forecasting framework for energy consumption and CO2 emissions. Appl. Energy 328, 120163. https://doi.org/10.1016/j.apenergy.2022.120163

Awang, N.R., Ramli, N.A., Yahaya, A.S., Elbayoumi, M. (2015). Multivariate methods to predict ground level ozone during daytime, nighttime, and critical conversion time in urban areas. Atmospheric Pollut. Res. 6, 726–734. https://doi.org/10.5094/APR.2015.081

Bai, Y., Dong, Y., Wang, W., Pan, D., Xu, Y., Zhong, Y., Chen, B., Chen, G., Wu, G., Wu, L., Wang, X., Hang, J. (2023). Air pollutant dispersion in street canyons based on an outdoor scale model and machine learning. Urban Clim. 47, 101381. https://doi.org/10.1016/j.uclim.2022.101381

Bonet, E.R., Do, T.H., Qin, X., Hofman, J., Manna, V.P.L., Philips, W., Deligiannis, N. (2022). Explaining Graph Neural Networks With Topology-Aware Node Selection: Application in Air Quality Inference. IEEE Trans. Signal Inf. Process. Netw. 8, 499–513. https://doi.org/10.1109/TSIPN.2022.3180679

Cabaneros, S.M., Calautit, J.K., Hughes, B. (2020). Spatial estimation of outdoor NO2 levels in Central London using deep neural networks and a wavelet decomposition technique. Ecol. Model. 424, 109017. https://doi.org/10.1016/j.ecolmodel.2020.109017

Cai, J., Ge, Y., Li, H., Yang, C., Liu, C., Meng, X., Wang, W., Niu, C., Kan, L., Schikowski, T., Yan, B., Chillrud, S.N., Kan, H., Jin, L. (2020). Application of land use regression to assess exposure and identify potential sources in PM2.5, BC, NO2 concentrations. Atmos. Environ. 223, 117267. https://doi.org/10.1016/j.atmosenv.2020.117267

Chen, Bin, Song, Z., Huang, J., Zhang, P., Hu, X., Zhang, X., Guan, X., Ge, J., Zhou, X. (2022a). Estimation of Atmospheric PM $_{10}$ Concentration in China Using an Interpretable Deep Learning Model and Top-of-the-Atmosphere Reflectance Data From China's New Generation Geostationary Meteorological Satellite, FY-4A. J. Geophys. Res. Atmospheres 127. https://doi.org/10.1029/2021JD036393

Chen, Bin, Song, Z., Pan, F., Huang, Y. (2022b). Obtaining vertical distribution of PM2.5 from CALIOP data and machine learning algorithms. Sci. Total Environ. 805, 150338. https://doi.org/10.1016/j.scitotenv.2021.150338

Chen, B., Song, Z., Shi, B., Li, M. (2022). An interpretable deep forest model for estimating hourly PM10 concentration in China using Himawari-8 data. Atmos. Environ. 268. https://doi.org/10.1016/j.atmosenv.2021.118827

Chen, B., Wang, Y., Huang, J., Zhao, L., Chen, R., Song, Z., Hu, J. (2023). Estimation of near-surface ozone concentration and analysis of main weather situation in China based on machine learning model and Himawari-8 TOAR data. Sci. Total Environ. 864, 160928. https://doi.org/10.1016/j.scitotenv.2022.160928

Chen, B., You, S., Ye, Y., Fu, Y., Ye, Z., Deng, J., Wang, K., Hong, Y. (2021a). An interpretable self-adaptive deep neural network for estimating daily spatially-continuous PM2.5 concentrations across China. Sci Total Env. 768, 144724. https://doi.org/10.1016/j.scitotenv.2020.144724

Chen, B., You, S., Ye, Y., Fu, Y., Ye, Z., Deng, J., Wang, K., Hong, Y. (2021b). An interpretable self-adaptive deep neural network for estimating daily spatially-continuous PM2.5 concentrations across China. SCIENCE OF THE TOTAL ENVIRONMENT. https://doi.org/10.1016/j.scitotenv.2020.144724

Cheng, Y., Huang, X.-F., Peng, Y., Tang, M.-X., Zhu, B., Xia, S.-Y., He, L.-Y. (2023). A novel machine learning method for evaluating the impact of emission sources on ozone formation. Environ. Pollut. 316, 120685. https://doi.org/10.1016/j.envpol.2022.120685

Choi, H.-S., Song, K., Kang, M., Kim, Y., Lee, K.-K., Choi, H. (2022a). Deep learning algorithms for prediction of PM10 dynamics in urban and rural areas of Korea. Earth Sci Inf. 15, 845–853. https://doi.org/10.1007/s12145-022-00771-1

Choi, H.-S., Song, K., Kang, M., Kim, Y., Lee, K.-K., Choi, H. (2022b). Deep learning algorithms for prediction of PM10 dynamics in urban and rural areas of Korea. EARTH SCIENCE INFORMATICS. https://doi.org/10.1007/s12145-022-00771-1

Coker, E.S., Amegah, A.K., Mwebaze, E., Ssematimba, J., Bainomugisha, E. (2021). A land use regression model using machine learning and locally developed low cost particulate matter sensors in Uganda. Environ. Res. 199, 111352. https://doi.org/10.1016/j.envres.2021.111352

De Hoogh, K., Héritier, H., Stafoggia, M., Künzli, N., Kloog, I. (2018). Modelling daily PM2.5 concentrations at high spatio-temporal resolution across Switzerland. Environ. Pollut. 233, 1147–1154. https://doi.org/10.1016/j.envpol.2017.10.025

Du, W., Chen, L., Wang, H., Shan, Z., Zhou, Z., Li, W., Wang, Y. (2023). Deciphering urban traffic impacts on air quality by deep learning and emission inventory. J. Environ. Sci. 124, 745–757. https://doi.org/10.1016/j.jes.2021.12.035

Eeftens, M., Odabasi, D., Flückiger, B., Davey, M., Ineichen, A., Feigenwinter, C., Tsai, M.-Y. (2019). Modelling the vertical gradient of nitrogen dioxide in an urban area. Sci. Total Environ. 650, 452–458. https://doi.org/10.1016/j.scitotenv.2018.09.039

Fung, P.L., Zaidan, M.A., Timonen, H., Niemi, J.V., Kousa, A., Kuula, J., Luoma, K., Tarkoma, S., Petäjä, T., Kulmala, M., Hussein, T. (2021). Evaluation of white-box versus black-box machine learning models in estimating ambient black carbon concentration. J Aerosol Sci 152, 105694. https://doi.org/10.1016/j.jaerosci.2020.105694

Gaeta, A., Cattani, G., Bucchianico, A.D.M. di, Santis, A.D., Cesaroni, G., Badaloni, C., Ancona, C., Forastiere, F., Sozzi, R., Bolignano, A., Sacco, F. (2016). Development of nitrogen dioxide and volatile organic compounds land use regression models to estimate air pollution exposure near an Italian airport. Atmos. Environ. 131, 254–262. https://doi.org/10.1016/j.atmosenv.2016.01.052

Gao, X., Li, W. (2021). A graph-based LSTM model for PM2.5 forecasting. Atmospheric Pollut. Res. 12, 101150. https://doi.org/10.1016/j.apr.2021.101150

García, M.V., Aznarte, J.L. (2020). Shapley additive explanations for NO2 forecasting. Ecol. Inform. 56, 101039. https://doi.org/10.1016/j.ecoinf.2019.101039

Ge, Y., Fu, Q., Yi, M., Chao, Y., Lei, X., Xu, X., Yang, Z., Hu, J., Kan, H., Cai, J. (2022). High spatial resolution land-use regression model for urban ultrafine particle exposure assessment in Shanghai, China. Sci. Total Environ. 816, 151633. https://doi.org/10.1016/j.scitotenv.2021.151633

Grange, S.K., Carslaw, D.C., Lewis, A.C., Boleti, E., Hueglin, C. (2018). Random forest meteorological normalisation models for Swiss PM₁₀ trend analysis. Atmospheric Chem. Phys. 18, 6223–6239. https://doi.org/10.5194/acp-18-6223-2018

Gryparis, A., Dimakopoulou, K., Pedeli, X., Katsouyanni, K. (2014). Spatio-temporal semiparametric models for NO2 and PM10 concentration levels in Athens, Greece. Sci. Total Environ. 479–480, 21–30. https://doi.org/10.1016/j.scitotenv.2014.01.075

Gu, J., Yang, B., Brauer, M., Zhang, K.M. (2021a). Enhancing the Evaluation and Interpretability of Data-Driven Air Quality Models. Atmos Env. 246, 118125. https://doi.org/10.1016/j.atmosenv.2020.118125

Gu, J., Yang, B., Brauer, M., Zhang, K.M. (2021b). Enhancing the Evaluation and Interpretability of Data-Driven Air Quality Models. ATMOSPHERIC ENVIRONMENT. https://doi.org/10.1016/j.atmosenv.2020.118125

Gu, Y., Li, B., Meng, Q. (2022). Hybrid interpretable predictive machine learning model for air pollution prediction. Neurocomputing 468, 123–136. https://doi.org/10.1016/j.neucom.2021.09.051

Habermann, M., Billger, M., Haeger-Eugensson, M. (2015). Land use Regression as Method to Model Air Pollution. Previous Results for Gothenburg/Sweden. Procedia Eng. 115, 21–28. https://doi.org/10.1016/j.proeng.2015.07.350

Han, L., Zhao, J., Gao, Y., Gu, Z. (2022). Prediction and evaluation of spatial distributions of ozone and urban heat island using a machine learning modified land use regression method. Sustain. Cities Soc. 78. https://doi.org/10.1016/j.scs.2021.103643

Hellack, B., Sugiri, D., Schins, R.P.F., Schikowski, T., Krämer, U., Kuhlbusch, T.A.J., Hoffmann, B. (2017). Land use regression modeling of oxidative potential of fine particles, NO2, PM2.5 mass and association to type two diabetes mellitus. Atmos. Environ. 171, 181–190. https://doi.org/10.1016/j.atmosenv.2017.10.017

Ho, C.-C., Chan, C.-C., Cho, C.-W., Lin, H.-I., Lee, J.-H., Wu, C.-F. (2015). Land use regression modeling with vertical distribution measurements for fine particulate matter and elements in an urban area. Atmos. Environ. 104, 256–263. https://doi.org/10.1016/j.atmosenv.2015.01.024

Hu, C., Kang, P., Jaffe, D.A., Li, C., Zhang, X., Wu, K., Zhou, M. (2021). Understanding the impact of meteorology on ozone in 334 cities of China. Atmos. Environ. 248, 118221. https://doi.org/10.1016/j.atmosenv.2021.118221

Huang, L., Zhang, C., Bi, J. (2017). Development of land use regression models for PM2.5, SO2, NO2 and O3 in Nanjing, China. Environ. Res. 158, 542–552. https://doi.org/10.1016/j.envres.2017.07.010

Iglesias-Gonzalez, S., Huertas-Bolanos, M.E., Hernandez-Paniagua, I.Y., Mendoza, A. (2020). Explicit Modeling of Meteorological Explanatory Variables in Short-Term Forecasting of Maximum Ozone Concentrations via a Multiple Regression Time Series Framework. J Atmos 11, 1304. https://doi.org/10.3390/atmos11121304

Jin, L., Berman, J.D., Warren, J.L., Levy, J.I., Thurston, G., Zhang, Yawei, Xu, X., Wang, S., Zhang, Yaqun, Bell, M.L. (2019). A land use regression model of nitrogen dioxide and fine particulate matter in a complex urban core in Lanzhou, China. Environ. Res. 177, 108597. https://doi.org/10.1016/j.envres.2019.108597

Jones, R.R., Hoek, G., Fisher, J.A., Hasheminassab, S., Wang, D., Ward, M.H., Sioutas, C., Vermeulen, R., Silverman, D.T. (2020). Land use regression models for ultrafine particles, fine particles, and black carbon in Southern California. Sci. Total Environ. 699, 134234. https://doi.org/10.1016/j.scitotenv.2019.134234

Jovanovic, Luka, Jovanovic, G., Perisic, M., Alimpic, F., Stanisic, S., Bacanin, N., Zivkovic, M., Stojic, A. (2023). The Explainable Potential of Coupling Metaheuristics-Optimized-XGBoost and SHAP in Revealing VOCs' Environmental Fate. J Atmos 14, 109. https://doi.org/10.3390/atmos14010109

Jovanovic, L., Jovanovic, G., Perisic, M., Alimpic, F., Stanisic, S., Bacanin, N., Zivkovic, M., Stojic, A. (2023). The Explainable Potential of Coupling Metaheuristics-Optimized-XGBoost and SHAP in Revealing VOCs' Environmental Fate. Atmosphere 14. https://doi.org/10.3390/atmos14010109

Just, A.C., Arfer, K.B., Rush, J., Dorman, M., Shtein, A., Lyapustin, A., Kloog, I. (2020). Advancing methodologies for applying machine learning and evaluating spatiotemporal models of fine particulate matter (PM2.5) using satellite data over large regions. Atmos. Environ. 239, 117649. https://doi.org/10.1016/j.atmosenv.2020.117649

Kang, Y., Choi, H., Im, J., Park, S., Shin, M., Song, C.-K., Kim, S. (2021). Estimation of surface-level NO2 and O3 concentrations using TROPOMI data and machine learning over East Asia. Environ. Pollut. 288. https://doi.org/10.1016/j.envpol.2021.117711

Ke, H., Gong, S., He, J., Zhang, L., Mo, J. (2022). A hybrid XGBoost-SMOTE model for optimization of operational air quality numerical model forecasts. Front. Environ. Sci. 10, 1007530. https://doi.org/10.3389/fenvs.2022.1007530

Kerckhoffs, J., Hoek, G., Portengen, L., Brunekreef, B., Vermeulen, R.C.H. (2019). Performance of Prediction Algorithms for Modeling Outdoor Air Pollution Spatial Surfaces. Environ. Sci. Technol. 53, 1413–1421. https://doi.org/10.1021/acs.est.8b06038

Kim, D., Ho, C.-H., Park, I., Kim, J., Chang, L.-S., Choi, M.-H. (2022a). Untangling the contribution of input parameters to an artificial intelligence PM2.5 forecast model using the layer-wise relevance propagation method. Atmos Env. 276, 119034. https://doi.org/10.1016/j.atmosenv.2022.119034

Kim, D., Ho, C.-H., Park, I., Kim, J., Chang, L.-S., Choi, M.-H. (2022b). Untangling the contribution of input parameters to an artificial intelligence PM2.5 forecast model using the layer-wise relevance propagation method. ATMOSPHERIC ENVIRONMENT. https://doi.org/10.1016/j.atmosenv.2022.119034

Kim, M., Brunner, D., Kuhlmann, G. (2021). Importance of satellite observations for high-resolution mapping of near-surface NO2 by machine learning. Remote Sens. Environ. 264, 112573. https://doi.org/10.1016/j.rse.2021.112573

Kleinert, F., Gong, B., Götz, M., Schultz, M.G. (2019). Near Surface Ozone Predictions Based on Multiple Artificial Neural Network Architectures 12541.

Kleinert, F., Leufen, L.H., Schultz, M.G. (2021). IntelliO3-ts v1.0: a neural network approach to predict near-surface ozone concentrations in Germany. Geosci. Model Dev. 14, 1–25. https://doi.org/10.5194/gmd-14-1-2021

Kumar, A., Goyal, P. (2011). Forecasting of daily air quality index in Delhi. Sci. Total Environ. 409, 5517–5523. https://doi.org/10.1016/j.scitotenv.2011.08.069

Lešnik, U., Mongus, D., Jesenko, D. (2019). Predictive analytics of PM 10 concentration levels using detailed traffic data. Transp Res Transp Env. 67, 131–141. https://doi.org/10.1016/j.trd.2018.11.015

Li, J., An, X., Li, Q., Wang, C., Yu, H., Zhou, X., Geng, Y. (2022). Application of XGBoost algorithm in the optimization of pollutant concentration. Atmospheric Res. 276, 106238. https://doi.org/10.1016/j.atmosres.2022.106238

Li, Y., Sun, Y. (2021). Modeling and predicting city-level CO2 emissions using open access data and machine learning. Environ. Sci. Pollut. Res. 28, 19260–19271. https://doi.org/10.1007/s11356-020-12294-7

Lin, S., Zhao, J., Li, J., Liu, X., Zhang, Y., Wang, S., Mei, Q., Chen, Z., Gao, Y. (2022). A Spatial–Temporal Causal Convolution Network Framework for Accurate and Fine-Grained PM2.5 Concentration Prediction. Entropy 24, 1125. https://doi.org/10.3390/e24081125

Liu, M., Chen, H., Wei, D., Wu, Y., Li, C. (2021). Nonlinear relationship between urban form and street-level PM2.5 and CO based on mobile measurements and gradient boosting decision tree models. Build. Environ. 205, 108265. https://doi.org/10.1016/j.buildenv.2021.108265

Liu, W., Li, X., Chen, Z., Zeng, G., León, T., Liang, J., Huang, G., Gao, Z., Jiao, S., He, X., Lai, M. (2015). Land use regression models coupled with meteorology to model spatial and temporal variability of NO2 and PM10 in Changsha, China. Atmos. Environ. 116, 272–280. https://doi.org/10.1016/j.atmosenv.2015.06.056

Liu, X., Zhao, J., Lin, S., Li, J., Wang, S., Zhang, Y., Gao, Y., Chai, J. (2022a). Fine-Grained Individual Air Quality Index (IAQI) Prediction Based on Spatial-Temporal Causal Convolution Network: A Case Study of Shanghai. J Atmos 13, 959. https://doi.org/10.3390/atmos13060959

Liu, X., Zhao, J., Lin, S., Li, J., Wang, S., Zhang, Y., Gao, Y., Chai, J. (2022b). Fine-Grained Individual Air Quality Index (IAQI) Prediction Based on Spatial-Temporal Causal Convolution Network: A Case Study of Shanghai. Atmosphere 13, 959. https://doi.org/10.3390/atmos13060959

Lovrić, M., Pavlović, K., Vuković, M., Grange, S.K., Haberl, M., Kern, R. (2021). Understanding the true effects of the COVID-19 lockdown on air pollution by means of machine learning. Environ. Pollut. 274, 115900. https://doi.org/10.1016/j.envpol.2020.115900

Lucena-Sánchez, E., Sciavicco, G., Stan, I.E. (2021). Feature and Language Selection in Temporal Symbolic Regression for Interpretable Air Quality Modelling. Algorithms 14, 76. https://doi.org/10.3390/a14030076

Luminati, O., Campos, B.L. de A. de, Flückiger, B., Brentani, A., Röösli, M., Fink, G., Hoogh, K. de (2021). Land use regression modelling of NO2 in São Paulo, Brazil. Environ. Pollut. 289, 117832. https://doi.org/10.1016/j.envpol.2021.117832

Luo, N., Zang, Z., Yin, C., Liu, M., Jiang, Y., Zuo, C., Zhao, W., Shi, W., Yan, X. (2022). Explainable and spatial dependence deep learning model for satellite-based O3 monitoring in China. Atmos. Environ. 290, 119370. https://doi.org/10.1016/j.atmosenv.2022.119370

Marvin, D., Nespoli, L., Strepparava, D., Medici, V. (2021). A data-driven approach to forecasting ground-level ozone concentration. Int. J. Forecast. https://doi.org/10.1016/j.ijforecast.2021.07.008

Masri, S., Hou, H., Dang, A., Yao, T., Zhang, L., Wang, T., Qin, Z., Wu, S., Han, B., Chen, J.-C. (JC), Chen, Y., Wu, J. (2019). Development of spatiotemporal models to predict ambient ozone and NOx concentrations in Tianjin, China. Atmos. Environ. 213, 37–46. https://doi.org/10.1016/j.atmosenv.2019.05.060

Meng, X., Hand, J.L., Schichtel, B.A., Liu, Y. (2018). Space-time trends of PM2.5 constituents in the conterminous United States estimated by a machine learning approach, 2005–2015. Environ. Int. 121, 1137–1147. https://doi.org/10.1016/j.envint.2018.10.029

Messier, K.P., Katzfuss, M. (2021). Scalable penalized spatiotemporal land-use regression for ground-level nitrogen dioxide. Ann. Appl. Stat. 15, 688–710. https://doi.org/10.1214/20-aoas1422

Michanowicz, D.R., Shmool, J.L.C., Cambal, L., Tunno, B.J., Gillooly, S., Hunt, M.J.O., Tripathy, S., Shields, K.N., Clougherty, J.E. (2016). A hybrid land use regression/line-source dispersion model for predicting intra-urban NO2. Transp. Res. Part Transp. Environ. 43, 181–191. https://doi.org/10.1016/j.trd.2015.12.007

Nabavi, S.O., Nölscher, A.C., Samimi, C., Thomas, C., Haimberger, L., Lüers, J., Held, A. (2021). Site-scale modeling of surface ozone in Northern Bavaria using machine learning algorithms, regional dynamic models, and a hybrid model. Environ. Pollut. Barking Essex 1987 268, 115736. https://doi.org/10.1016/j.envpol.2020.115736

Naughton, O., Donnelly, A., Nolan, P., Pilla, F., Misstear, B.D., Broderick, B. (2018). A land use regression model for explaining spatial variation in air pollution levels using a wind sector based approach. Sci. Total Environ. 630, 1324–1334. https://doi.org/10.1016/j.scitotenv.2018.02.317
Park, Y., Kwon, B., Heo, J., Hu, X., Liu, Y., Moon, T. (2020). Estimating PM2.5 concentration of the conterminous United States via interpretable convolutional neural networks. Environ. Pollut. Barking Essex 1987 256, 113395. https://doi.org/10.1016/j.envpol.2019.113395
Peng, Z., Zhang, C., Cao, B., Hong, Z., Han, X. (2022). An interpretable prediction of FCM driven by small samples for energy analysis based on air quality prediction. J. Air Waste Manag. Assoc. 72, 985–999. https://doi.org/10.1080/10962247.2022.2064006
Qi, M., Hankey, S. (2021). Using Street View Imagery to Predict Street-Level Particulate Air Pollution. Environ. Sci. Technol. 55, 2695–2704. https://doi.org/10.1021/acs.est.0c05572
Ren, X., Mi, Z., Cai, T., Nolte, C.G., Georgopoulos, P.G. (2022). Flexible Bayesian Ensemble Machine Learning Framework for Predicting Local Ozone Concentrations. Environ. Sci. Technol. 56, 3871–3883. https://doi.org/10.1021/acs.est.1c04076
Ren, X., Mi, Z., Georgopoulos, P.G. (2020). Comparison of Machine Learning and Land Use Regression for fine scale spatiotemporal estimation of ambient air pollution: Modeling ozone concentrations across the contiguous United States. Environ. Int. 142, 105827. https://doi.org/10.1016/j.envint.2020.105827
Ronquillo-Lomeli, G., Rodríguez-Olivares, N.A., Barriga-Rodríguez, L., Ramírez-Martínez, A., Soto-Cajiga, J.A., Nava-Balanzar, L. (2022). Nonlinear modeling of industrial boiler NOx emissions. J. Air Waste Manag. Assoc. 72, 556–569. https://doi.org/10.1080/10962247.2021.1980451
Sadeghi, B., Ghahremanloo, M., Mousavinezhad, S., Lops, Y., Pouyaei, A., Choi, Y. (2022). Contributions of meteorology to ozone variations: Application of deep learning and the Kolmogorov-Zurbenko filter. Environ. Pollut. 310, 119863. https://doi.org/10.1016/j.envpol.2022.119863
Sanchez, M., Ambros, A., Milà, C., Salmon, M., Balakrishnan, K., Sambandam, S., Sreekanth, V., Marshall, J.D., Tonne, C. (2018). Development of land-use regression models for fine particles and black carbon in peri-urban South India. Sci. Total Environ. 634, 77–86. https://doi.org/10.1016/j.scitotenv.2018.03.308
Shi, H., Yang, N., Yang, X., Tang, H. (2023a). Clarifying Relationship between PM2.5 Concentrations and Spatiotemporal Predictors Using Multi-Way Partial Dependence Plots. Remote Sens 15, 358. https://doi.org/10.3390/rs15020358
Shi, H., Yang, N., Yang, X., Tang, H. (2023b). Clarifying Relationship between PM2.5 Concentrations and Spatiotemporal Predictors Using Multi-Way Partial Dependence Plots. REMOTE SENSING. https://doi.org/10.3390/rs15020358
Shi, T., Dirienzo, N., Requia, W.J., Hatzopoulou, M., Adams, M.D. (2020a). Neighbourhood scale nitrogen dioxide land use regression modelling with regression kriging in an urban transportation corridor. Atmos. Environ. 223, 117218. https://doi.org/10.1016/j.atmosenv.2019.117218
Shi, T., Hu, Y., Liu, M., Li, C., Zhang, C., Liu, C. (2020b). Land use regression modelling of PM2.5 spatial variations in different seasons in urban areas. Sci. Total Environ. 743, 140744. https://doi.org/10.1016/j.scitotenv.2020.140744
Song, S., Bang, S., Cho, S., Han, H., Lee, S. (2022). Attentive Multi-Task Prediction of Atmospheric Particulate Matter: Effect of the COVID-19 Pandemic. IEEE Access 10, 10176–10190. https://doi.org/10.1109/ACCESS.2022.3144588
Stadtler, S., Betancourt, C., Roscher, R. (2022). Explainable Machine Learning Reveals Capabilities, Redundancy, and Limitations of a Geospatial Air Quality Benchmark Dataset. MACHINE LEARNING AND KNOWLEDGE EXTRACTION. https://doi.org/10.3390/make4010008
Steininger, M., Kobs, K., Zehe, A., Lautenschlager, F., Becker, M., Hotho, A. (2020). MapLUR: Exploring a New Paradigm for Estimating Air Pollution Using Deep Learning on Map Images. ACM Trans. Spat. Algorithms Syst. 6. https://doi.org/10.1145/3380973
Stirnberg, R., Cermak, J., Kotthaus, S., Haeffelin, M., Andersen, H., Fuchs, J., Kim, M., Petit, J.-E., Favez, O. (2021). Meteorology-driven variability of air pollution (PM1) revealed with explainable machine learning. ATMOSPHERIC CHEMISTRY AND PHYSICS. https://doi.org/10.5194/acp-21-3919-2021
Sun, Y., Wang, X., Ren, N., Liu, Y., You, S. (2023). Improved Machine Learning Models by Data Processing for Predicting Life-Cycle Environmental Impacts of Chemicals. Environ. Sci. Technol. 57, 3434–3444. https://doi.org/10.1021/acs.est.2c04945
Sun, Z., Archibald, A.T. (2021). Multi-stage ensemble-learning-based model fusion for surface ozone simulations: A focus on CMIP6 models. Environ. Sci. Ecotechnology 8, 100124. https://doi.org/10.1016/j.ese.2021.100124
Tripathy, S., Tunno, B.J., Michanowicz, D.R., Kinnee, E., Shmool, J.L.C., Gillooly, S., Clougherty, J.E. (2019). Hybrid land use regression modeling for estimating spatio-temporal exposures to PM2.5, BC, and metal components across a metropolitan area of complex terrain and industrial sources. Sci. Total Environ. 673, 54–63. https://doi.org/10.1016/j.scitotenv.2019.03.453
Tularam, H., Ramsay, L.F., Muttoo, S., Brunekreef, B., Meliefste, K., Hoogh, K. de, Naidoo, R.N. (2021). A hybrid air pollution / land use regression model for predicting air pollution concentrations in Durban, South Africa. Environ. Pollut. 274, 116513. https://doi.org/10.1016/j.envpol.2021.116513
Varde, A.S., Pandey, A., Du, X. (2022). Prediction Tool on Fine Particle Pollutants and Air Quality for Environmental Engineering. SN Comput. Sci. 3, 184. https://doi.org/10.1007/s42979-022-01068-2
Vettori, S., Huser, R., Genton, M.G. (2019). Bayesian modeling of air pollution extremes using nested multivariate max-stable processes. Biometrics 75, 831–841. https://doi.org/10.1111/biom.13051
Wang, A., Xu, J., Tu, R., Saleh, M., Hatzopoulou, M. (2020a). Potential of machine learning for prediction of traffic related air pollution. Transp Res Trans Env. 88, 102599. https://doi.org/10.1016/j.trd.2020.102599

Wang, A., Xu, J., Tu, R., Saleh, M., Hatzopoulou, M. (2020b). Potential of machine learning for prediction of traffic related air pollution. TRANSPORTATION RESEARCH PART D-TRANSPORT AND ENVIRONMENT. https://doi.org/10.1016/j.trd.2020.102599

Wang, J., He, L., Lu, X., Zhou, L., Tang, H., Yan, Y., Ma, W. (2022). A full-coverage estimation of PM2.5 concentrations using a hybrid XGBoost-WD model and WRF-simulated meteorological fields in the Yangtze River Delta Urban Agglomeration, China. Env. Res 203, 111799. https://doi.org/10.1016/j.envres.2021.111799

Wang, L., Zhao, Y., Shi, J., Ma, J., Liu, X., Han, D., Gao, H., Huang, T. (2023). Predicting ozone formation in petrochemical industrialized Lanzhou city by interpretable ensemble machine learning. Environ. Pollut. 318, 120798. https://doi.org/10.1016/j.envpol.2022.120798

Wang, Y., Hu, X., Chang, H., Waller, L., Belle, J., Liu, Y. (2018). A Bayesian Downscaler Model to Estimate Daily PM2.5 Levels in the Conterminous US. Int. J. Environ. Res. Public. Health 15, 1999. https://doi.org/10.3390/ijerph15091999

Wei, Ning, Jia, Z., Men, Z., Ren, C., Zhang, Y., Peng, J., Wu, L., Wang, T., Zhang, Q., Mao, H. (2022). Machine Learning Predicts Emissions of Brake Wear PM $_{2.5}$: Model Construction and Interpretation. Environ. Sci. Technol. Lett. 9, 352–358. https://doi.org/10.1021/acs.estlett.2c00117

Wei, N., Zhang, Q., Zhang, Y., Jin, J., Chang, J., Yang, Z., Ma, C., Jia, Z., Ren, C., Wu, L., Peng, J., Mao, H. (2022). Super-learner model realizes the transient prediction of CO2 and NOx of diesel trucks: Model development, evaluation and interpretation. Environ. Int. 158. https://doi.org/10.1016/j.envint.2021.106977

Wu, C.-D., Chen, Y.-C., Pan, W.-C., Zeng, Y.-T., Chen, M.-J., Guo, Y.L., Lung, S.-C.C. (2017). Land-use regression with long-term satellite-based greenness index and culture-specific sources to model PM2.5 spatial-temporal variability. Environ. Pollut. 224, 148–157. https://doi.org/10.1016/j.envpol.2017.01.074

Wu, C.-F., Lin, H.-I., Ho, C.-C., Yang, T.-H., Chen, C.-C., Chan, C.-C. (2014). Modeling horizontal and vertical variation in intraurban exposure to PM2.5 concentrations and compositions. Environ. Res. 133, 96–102. https://doi.org/10.1016/j.envres.2014.04.038

Wu, Y., Lin, S., Shi, K., Ye, Z., Fang, Y. (2022a). Seasonal prediction of daily PM2.5 concentrations with interpretable machine learning: a case study of Beijing, China. Env. Sci Pollut Res 29, 45821–45836. https://doi.org/10.1007/s11356-022-18913-9

Wu, Y., Lin, S., Shi, K., Ye, Z., Fang, Y. (2022b). Seasonal prediction of daily PM2.5 concentrations with interpretable machine learning: a case study of Beijing, China. ENVIRONMENTAL SCIENCE AND POLLUTION RESEARCH. https://doi.org/10.1007/s11356-022-18913-9

Xie, C., Xie, Y., Zhang, C., Dong, H., Zhang, L. (2023). Explainable machine learning for carbon dioxide adsorption on porous carbon. J. Environ. Chem. Eng. 11, 109053. https://doi.org/10.1016/j.jece.2022.109053

Xu, J., Yang, W., Bai, Z., Zhang, R., Zheng, J., Wang, M., Zhu, T. (2022). Modeling spatial variation of gaseous air pollutants and particulate matters in a Metropolitan area using mobile monitoring data. Environ. Res. 210, 112858. https://doi.org/10.1016/j.envres.2022.112858

Yan, X., Zang, Z., Jiang, Y., Shi, W., Guo, Y., Li, D., Zhao, C., Husi, L. (2021a). A Spatial-Temporal Interpretable Deep Learning Model for improving interpretability and predictive accuracy of satellite-based PM2.5. Env. Pollut 273, 116459. https://doi.org/10.1016/j.envpol.2021.116459

Yan, X., Zang, Z., Jiang, Y., Shi, W., Guo, Y., Li, D., Zhao, C., Husi, L. (2021b). A Spatial-Temporal Interpretable Deep Learning Model for improving interpretability and predictive accuracy of satellite-based PM2.5. ENVIRONMENTAL POLLUTION. https://doi.org/10.1016/j.envpol.2021.116459

Yan, X., Zang, Z., Luo, N., Jiang, Y., Li, Z. (2020). New interpretable deep learning model to monitor real-time PM2.5 concentrations from satellite data. Environ. Int. 144, 106060. https://doi.org/10.1016/j.envint.2020.106060

Yang, N., Shi, H., Tang, H., Yang, X. (2022). Geographical and temporal encoding for improving the estimation of PM2.5 concentrations in China using end-to-end gradient boosting. Remote Sens. Environ. 269, 112828. https://doi.org/10.1016/j.rse.2021.112828

Yang, Y., Mei, G., Izzo, S. (2022). Revealing Influence of Meteorological Conditions on Air Quality Prediction Using Explainable Deep Learning. IEEE Access 10, 50755–50773. https://doi.org/10.1109/ACCESS.2022.3173734

Yin, X., Franklin, M., Fallah-Shorshani, M., Shafer, M., McConnell, R., Fruin, S. (2022). Exposure models for particulate matter elemental concentrations in Southern California. Environ. Int. 165, 107247. https://doi.org/10.1016/j.envint.2022.107247

Yu, M., Masrur, A., Blaszczak-Boxe, C. (2023a). Predicting hourly PM2.5 concentrations in wildfire-prone areas using a SpatioTemporal Transformer model. Sci Total Env. 860, 160446. https://doi.org/10.1016/j.scitotenv.2022.160446

Yu, M., Masrur, A., Blaszczak-Boxe, C. (2023b). Predicting hourly PM2.5 concentrations in wildfire-prone areas using a SpatioTemporal Transformer model. SCIENCE OF THE TOTAL ENVIRONMENT. https://doi.org/10.1016/j.scitotenv.2022.160446

Zaidan, M.A., Wraith, D., Boor, B.E., Hussein, T. (2019). Bayesian Proxy Modelling for Estimating Black Carbon Concentrations using White-Box and Black-Box Models. Appl. Sci. 9, 4976. https://doi.org/10.3390/app9224976

Zang, Z., Guo, Y., Jiang, Y., Zuo, C., Li, D., Shi, W., Yan, X. (2021). Tree-based ensemble deep learning model for spatiotemporal surface ozone (O3) prediction and interpretation. Int. J. Appl. Earth Obs. Geoinformation 103, 102516. https://doi.org/10.1016/j.jag.2021.102516

Zhai, B., Chen, J. (2018). Development of a stacked ensemble model for forecasting and analyzing daily average PM2.5 concentrations in Beijing, China. Sci. Total Environ. 635, 644–658. https://doi.org/10.1016/j.scitotenv.2018.04.040

Zhang, J.J.Y., Sun, L., Barrett, O., Bertazzon, S., Underwood, F.E., Johnson, M. (2015). Development of land-use regression models for metals associated with airborne particulate matter in a North American city. Atmos. Environ. 106, 165–177. https://doi.org/10.1016/j.atmosenv.2015.01.008

Zhang, J.J.Y., Sun, L., Rainham, D., Dummer, T.J.B., Wheeler, A.J., Anastasopolos, A., Gibson, M., Johnson, M. (2022). Predicting intraurban airborne PM1.0-trace elements in a port city: Land use regression by ordinary least squares and a machine learning algorithm. Sci. Total Environ. 806, 150149. https://doi.org/10.1016/j.scitotenv.2021.150149
Zhang, S., Mi, T., Wu, Q., Luo, Y., Grieneisen, M.L., Shi, G., Yang, F., Zhan, Y. (2022a). A data-augmentation approach to deriving long-term surface SO2 across Northern China: Implications for interpretable machine learning. Sci Total Env. 827, 154278. https://doi.org/10.1016/j.scitotenv.2022.154278
Zhang, S., Mi, T., Wu, Q., Luo, Y., Grieneisen, M.L., Shi, G., Yang, F., Zhan, Y. (2022b). A data-augmentation approach to deriving long-term surface SO2 across Northern China: Implications for interpretable machine learning. SCIENCE OF THE TOTAL ENVIRONMENT. https://doi.org/10.1016/j.scitotenv.2022.154278
Zhang, Z., Xu, B., Xu, W., Wang, F., Gao, J., Li, Y., Li, M., Feng, Y., Shi, G. (2022). Machine learning combined with the PMF model reveal the synergistic effects of sources and meteorological factors on PM2.5 pollution. Environ. Res. 212, 113322. https://doi.org/10.1016/j.envres.2022.113322
Zhou, H., Zhang, F., Du, Z., Liu, R. (2022a). A theory-guided graph networks based PM2.5 forecasting method. Environ. Pollut. 293, 118569. https://doi.org/10.1016/j.envpol.2021.118569
Zhou, H., Zhang, F., Du, Z., Liu, R. (2022b). A theory-guided graph networks based PM2.5 forecasting method. Environ. Pollut. 293, 118569. https://doi.org/10.1016/j.envpol.2021.118569
Zhu, J., Deng, F., Zhao, J., Zheng, H. (2021). Attention-based parallel networks (APNet) for PM2.5 spatiotemporal prediction. Sci. Total Environ. 769, 145082. https://doi.org/10.1016/j.scitotenv.2021.145082
